# Genetic risk, metabolic syndrome, and gastrointestinal cancer risk: A prospective cohort study

**DOI:** 10.1002/cam4.4923

**Published:** 2022-06-22

**Authors:** Yaqian Liu, Caiwang Yan, Shuangshuang Yin, Tianpei Wang, Meng Zhu, Li Liu, Guangfu Jin

**Affiliations:** ^1^ Department of Epidemiology, Center for Global Health, School of Public Health Nanjing Medical University Nanjing China; ^2^ Nanjing Drum Tower Hospital Clinical College of Nanjing Medical University Nanjing China; ^3^ Jiangsu Key Lab of Cancer Biomarkers, Prevention and Treatment, Collaborative Innovation Center for Cancer Personalized Medicine and China International Cooperation Center for Environment and Human Health Nanjing Medical University Nanjing China; ^4^ Digestive Endoscopy Department and General Surgery Department The First Affiliated Hospital with Nanjing Medical University and Jiangsu Province Hospital Nanjing China

**Keywords:** gastrointestinal cancer, genetic risk, metabolic syndrome, polygenic risk score

## Abstract

**Background:**

Gastrointestinal (GI) cancer risk has been associated with metabolic syndrome (MetS), a surrogate indicator for unhealthy lifestyles, and a number of genetic loci, but the combined effect of MetS and genetic variants on GI cancer risk is uncertain.

**Methods:**

We included 430,036 participants with available MetS and genotype data from UK Biobank. During the follow‐up time, 5494 incident GI cancer cases, including esophageal cancer, gastric cancer, and colorectal cancer, were identified. We created a GI polygenic risk score (GI‐PRS) for overall GI cancer derived from three site‐specific cancer PRSs. Cox proportional hazards regression was used to estimate the associations of MetS and GI‐PRS with the risk of GI cancer.

**Results:**

MetS was significantly associated with 28% increment in GI cancer risk (hazard ratio [HR]_MetS vs. non‐MetS_: 1.28, 95% confidence interval [CI]: 1.21–1.35, *p* < 0.0001), whereas a high GI‐PRS (top quintile) was associated with 2.28‐fold increase in risk (HR_high vs. low_: 2.28, 95% CI: 2.09–2.49, *p* < 0.0001). Compared with participants without MetS and at low genetic risk (bottom quintile of GI‐PRS), those with MetS and at high genetic risk had 2.75‐fold increase in GI cancer risk (HR: 2.75, 95% CI: 2.43–3.12, *p* < 0.0001). Additionally, MetS in comparison with no MetS had 1.49‰, 2.75‰, and 3.37‰ absolute risk increases in 5 years among participants at low, intermediate (quintiles 2–4 of GI‐PRS) and high genetic risk, respectively, representing the number of subjects diagnosed as MetS causing a new GI cancer case in 5 years were 669, 364, and 296, respectively.

**Conclusions:**

Metabolic and genetic factors may jointly contribute to GI cancer risk and may serve as predictors by quantitative measurements to identify high‐risk populations of GI cancer for precise prevention.

## Graphical abstract

We developed a composite polygenic risk score to measure genetic risk for overall gastrointestinal (GI) cancer and then analyzed the joint effect of metabolic syndrome and genetic factors on GI cancer risk based on a large‐scale cohort study. Our results show that metabolic and genetic factors jointly contributed to 2.75‐fold increased risk of GI cancer, which means they may be incorporated into predictive models to identify high risk population of GI cancer for precise prevention.

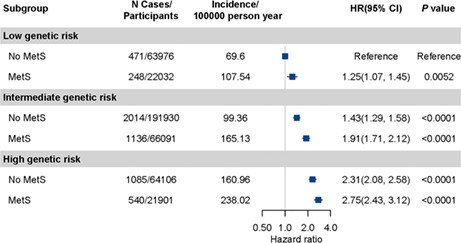

## 1 INTRODUCTION

Gastrointestinal (GI) cancer, including esophageal cancer (ESC), gastric cancer (GC) and colorectal cancer (CRC), is among the top 10 causes of cancer morbidity and mortality worldwide.^1^ Both environmental and genetic factors are known to play a role in the development of GI cancer. Metabolic syndrome (MetS), a cluster of metabolic risk factors that includes central obesity, insulin resistance, dyslipidemia, and hypertension, has been a global public health and a clinical issue.^2^ It is well established that MetS is linked to an increased risk of cardiovascular diseases and Type 2 diabetes.^3^ The components of MetS were also documented as risk factors for many cancers,^4^, ^5^, ^6^, ^7^ and previous observational studies indicated that participants with MetS showed higher risk for esophageal adenocarcinoma^8^ and colorectal cancer.^9^ Therefore, MetS may represent a metabolic indicator to predict GI cancer risk.

Recently, many studies have provided insight into the joint effect of known lifestyle or clinical risk factors and genetic factors on site‐specific and overall cancers.^10^, ^11^, ^12^, ^13^ Dozens of susceptibility loci associated with the risk of ESC, GC, and CRC have been found by genome‐wide association studies (GWASs).^14^, ^15^, ^16^ Although these loci showed relatively modest effects on cancer risk individually, polygenic risk scores (PRSs) integrating numerous loci have been proven to effectively predict incident cases of ESC, GC, and CRC, respectively.^10^, ^14^, ^17^ Since individual germline variants do not change over time, PRS could not be just confined to the risk stratification of site‐specific GI cancer. Therefore, PRS may act as a genetic indicator to measure genetic risk of cancer, including overall GI cancer.

In this study, we constructed a polygenetic risk score for overall GI cancer (GI‐PRS) derived from individual PRSs of ESC, GC, and CRC, and then investigated the association of MetS and GI‐PRS with GI cancer risk among participants in UK Biobank (UKB). The findings of our study may contribute to identifying populations at high‐risk of GI cancer for personalized prevention and screening.

## 2 MATERIAL AND METHODS

### 2.1 Study population

The UKB is a large prospective cohort study comprising 502,447 participants with genetic and phenotypic data. These participants were aged between 40 and 69 and recruited from 22 centers across England, Wales, and Scotland between 2006 and 2010.^18^, ^19^ The study has approval from the North West Multi‐Centre Research Ethics Committee, the National Information Governance Board for Health and Social Care in England and Wales, and the Community Health Index Advisory Group in Scotland and each eligible participant provided written informed consent.

Before analysis, we excluded participants with prevalent cancer at baseline (except non‐melanoma skin cancer [C44], *n* = 29,721); those having missing information on all MetS components (*n* = 628); women pregnant at recruitment due to their larger waist circumference and possible metabolic changes during pregnancy^20^ (*n* = 148); nonwhite people (*n* = 28,381); those whose genetic sex differed from reported gender (*n* = 313) or whose genotype data were unavailable (*n* = 12,421); cases diagnosed within the first year of follow‐up to reduce the impact of reverse causality bias^21^ (*n* = 447) or those having been diagnosed with other cancers before the diagnosis of GI cancer to avoid false association since other cancers may increase risk of GI cancer^22^ (*n* = 352) (Figure S1).

### 2.2 Exposure and covariate ascertainment

During baseline recruitment, comprehensive phenotype information, including sociodemographic, lifestyle exposures, and other health information, was collected using a touch‐screen questionnaire. Participants also underwent comprehensive physical measurements like waist circumference, body mass index (BMI). Blood pressure, including systolic and diastolic blood pressure (mmHg), were measured twice after participants rested for five or more minutes, and the mean of the two measures was used. Furthermore, blood samples were collected for genotyping and concentration measurements of serum triglycerides, high density lipoproteins, glucose, and glycated hemoglobin (HbA1c). To address missing values, sex‐specific medians were imputed for continuous variables and a missing category (including “do not know” or “prefer not to answer”) was added for each categorical variable.

### 2.3 Follow‐up and outcome assessment

Primary diagnosis of malignant cancers within the UKB was ascertained through records linkage with national cancer and death registries. We focused on incident GI cancers coded by the 10th Revision of the International Classification of Diseases (ICD‐10), including esophagus (C15), stomach (C16), and colorectum (C18–20). Follow‐up ended at the date of first‐ever any GI cancer diagnosis, death, loss to follow‐up or the end of follow‐up on 31 October 2015 for Scotland and 29 February 2020 for England and Wales, whichever came first.

### 2.4 Definition of MetS

MetS is defined as having any three or more of five MetS components, including central obesity, hypertension, dyslipidemia, hypertriglyceridemia, and hyperglycemia, according to the National Cholesterol Education Programme Adult Treatment Panel III criteria (NCEP‐ATP III).^23^ Details about MetS definition are shown in Table S1. Since fasting blood glucose level was not available in the cohort, HbA1c was chosen as a surrogate with a cutoff of ≥42 mmol/mol (≥6%).^24^

### 2.5 Polygenic risk score

Detailed descriptions of genotyping process for the single nucleotide polymorphisms (SNPs) in the UKB study have been provided elsewhere.^18^, ^25^ Summary association statistics were derived from external GWASs of site‐specific GI cancers with the largest sample size in European ancestry by January 1, 2020.^14^, ^15^, ^16^ Variants with *p* < 5 × 10^−8^ and minor allele frequency (MAF) ≥0.01 were extracted from eligible GWASs. For variants that were not available in the UKB, their strong correlated SNPs (*r* ^2^ > 0.8) were selected as a surrogate. We removed SNPs with MAF differences >0.10 or allele mismatches with reference to the 1000 Genomes European population, as well as palindromic SNPs (A/T, G/C) with MAF ≥0.45. If multiple correlated SNPs in the same locus were reported, those with the lowest reported *p* value were selected by using the linkage disequilibrium (LD) clumping procedure (at *r* ^2^ < 0.2) in PLINK. Finally, 13, 3, and 90 SNPs for ESC, GC, and CRC were included, respectively (Figure S2; Table S2). Site‐specific PRSs for each participant were calculated by summing the genotype dosage of each risk allele after multiplication with its respective effect size. No SNPs were shared or in high LD (*r* ^2^ > 0.6) with each other in more than one site‐specific PRSs. Then, we built a GI‐PRS to assess overall GI cancer risk by summing site‐specific PRSs weighted by their respective age‐standardized incidence rate in UK population.^26^, ^27^ The GI‐PRS was divided into three levels of genetic risk: Low (lowest quintile), moderate (quintiles 2–4), and high (top quintile).

### 2.6 Statistical analysis

The effect of MetS and GI‐PRS on GI cancer was estimated using Cox proportional hazards models, which yielded hazard ratios (HRs) and 95% confidence intervals (CIs) based on follow‐up time from baseline. The proportional hazards assumptions were tested using Schoenfeld Residuals. Analyses were adjusted for the known risk factors for GI cancer, including age group at baseline, gender, qualification, Townsend Deprivation Index, family history of cancer, physical activity, smoking status, alcohol consumption, fruit intake, vegetable consumption, red and processed meat consumption,^28^ and regular aspirin or ibuprofen use (Table S3). We additionally adjusted the top 10 genetic principal components of ancestry in the models including genetic risk. Also, we used an interaction term in the model to assess the statistical interaction between genetic risk and MetS. Absolute risk was evaluated as the percentage of incident GI cancer cases for a given group and absolute risk increase was estimated as the difference in absolute risk in 5‐year event rates among given groups. We also extrapolated the number of subjects with MetS needed to cause a new GI cancer case in 5 years for the given groups. The 95% CIs were calculated using 1000 bootstrap samples drawn from the estimation dataset.

We further adjusted for five MetS components to test the independent effect of each MetS component on GI cancer risk. Subgroup analyses were conducted to assess potential modification effects. The relationship between GI‐PRS and MetS risk was evaluated using a multivariable logistic regression model. The potentially nonlinear association between GI‐PRS and GI cancer risk was modeled using restricted cubic spline analysis. To assess the robustness of our findings, we performed several sensitivity analyses: (i) Defining obesity as BMI >30 kg/m^2^ instead of waist circumference in metabolic status; (ii) censoring incident cases within the first 2 years of follow up; (iii) validating the main results with unimputed data; (iv) repeating the analysis with unrelated participants by excluding those identified with at least one relative; (v) reconstructing GI‐PRS by standardizing the mean of each site‐specific cancer PRS to 1. All statistical tests were two‐sided, with statistical significance defined as *p* < 0.05. R software (version 3.6.3; R Project for Statistical Computing) was used to conduct all statistical analyses.

## 3 RESULTS

In the final analysis, a total of 430,036 eligible participants, including 199,520 (46.40%) men and 230,516 (53.60%) women, were included. Within a median follow‐up of 10.9 (interquartile range: 10.0–11.5) years, 5494 incident GI cancers (782 ESC, 516 GC, and 4205 CRC) were diagnosed. Baseline characteristics are presented in Table S4, while characteristics of each cancer are presented in Table S5.

**FIGURE 1:**
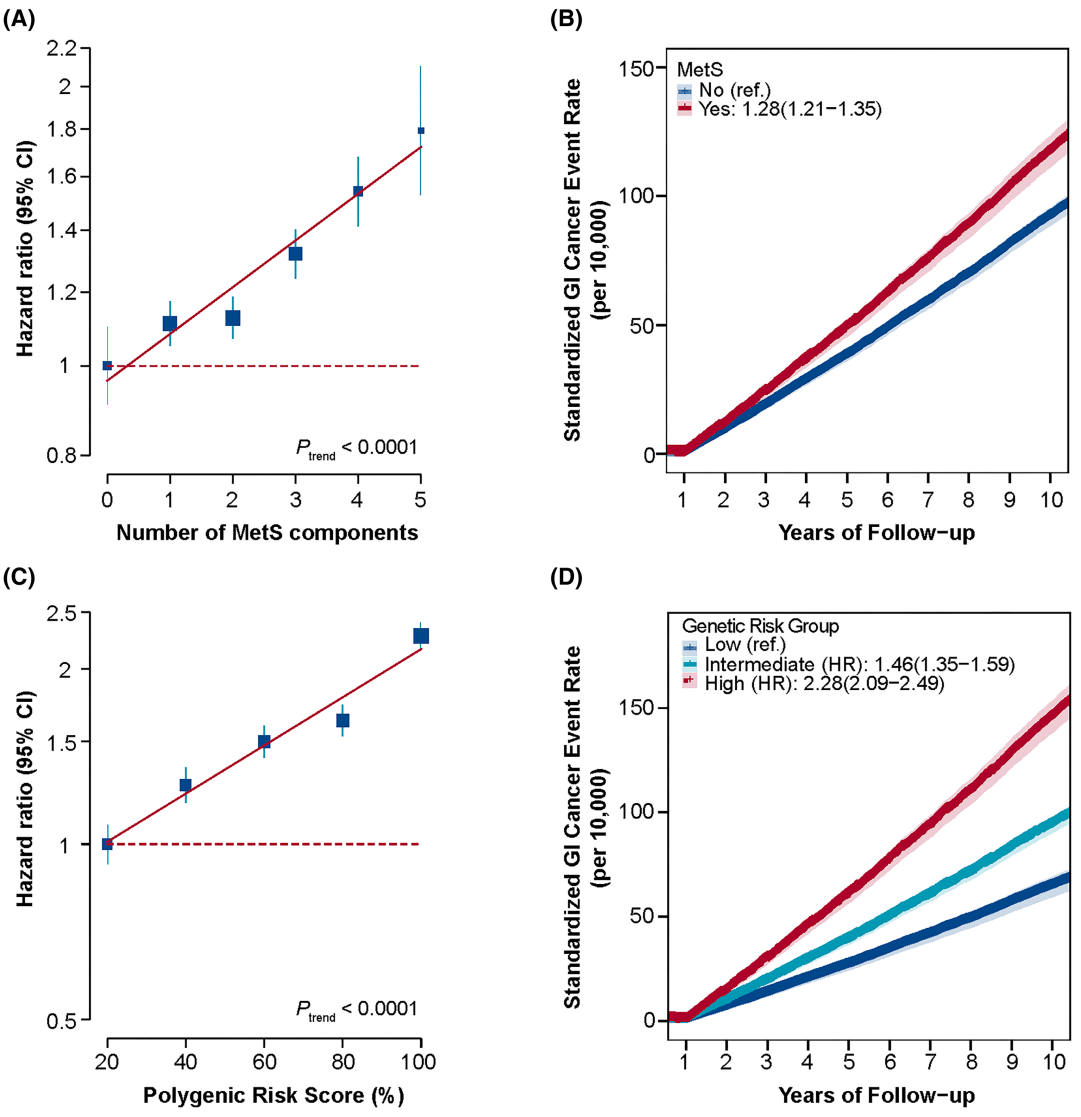
The relationship of MetS and PRS with GI cancer risk. (A) Participants were divided into six groups based on the number of MetS components they had and HR of each group was compared to participants having no MetS components. (B) Standardized rates of GI cancer events in non‐MetS and MetS groups. (C) Participants were grouped based on PRS quintiles and HR of each group was compared to those at the lowest quintile. (D) Standardized rates of GI cancer events in those at low (lowest quintile), intermediate (quintiles 2–4), and high (top quintile) genetic risk. GI, gastrointestinal; HR, hazard ratio; MetS, metabolic syndrome; PRS, polygenic risk score.

According to the NCEP‐ATP III criteria, 110,024 (25.58%) participants were classified as MetS. Compared with participants without GI cancers, incident GI cancer cases had higher level of MetS components and were more likely to be diagnosed as MetS at baseline (Table 1). Correspondingly, the risk of GI cancer increased as the number of unfavorable MetS components increased (*p* < 0.0001) (Figure 1A). MetS was associated with a high‐risk of overall GI cancer in both the minimally (HR = 1.31, 95% CI: 1.23–1.38, *p* < 0.0001) and fully adjusted model (HR = 1.28, 95% CI: 1.21–1.35, *p* < 0.0001) (Table 2; Figure 1B). When compared to participants without any unfavorable MetS components, GI cancer risk for those with three or more components was 1.41‐fold higher (HR = 1.41, 95% CI: 1.27–1.56, *p* < 0.0001) (Table 2). Furthermore, we found that only central obesity and hyperglycemia independently increased GI cancer risk after internal adjustment for all components, no matter in dichotomy or continuity (Tables S6 and S7). Similar association patterns were observed between MetS and risk of ESC, GC and CRC (Table S8; Figure S3). Results from sensitive analyses were generally consistent with those from main analysis (Tables S9–S11; Figure S4). In the subgroup analyses, similar association effects of MetS with GI cancer risk were observed between subgroups by gender, smoking status, alcohol consumption, physical activity, fruit intake, vegetable consumption, red and processed meat consumption, and regular aspirin or ibuprofen use except age (Figure S5).

**TABLE 1:** Descriptive statistics of metabolic and genetic factors of participants from UK Biobank

Characteristics	All (*N* = 430,036)	Cases (*N* = 5494)	Noncases (*N* = 424,542)
Waist circumference, Mean (SD), cm	90.27 (13.49)	94.57 (13.95)	90.21 (13.48)
SBP, Mean (SD), mmHg	138 (18.07)	142 (18.68)	138 (18.06)
DBP, Mean (SD), mmHg	82 (9.84)	83 (9.99)	82 (9.84)
Triglycerides, Mean (SD), mmol/L	1.74 (1.00)	1.89 (1.05)	1.74 (1.00)
HDL, Mean (SD), mmol/L	1.45 (0.36)	1.39 (0.36)	1.45 (0.36)
HbA1c, Mean (SD), mmol/mol	35.88 (6.35)	37.16 (7.15)	35.86 (6.34)
MetS (%)			
No	320,012 (74.42)	3570 (64.98)	316,442 (74.54)
Yes	110,024 (25.58)	1924 (35.02)	108,100 (25.46)
GI‐PRS (%)			
Low	86,008 (20.00)	719 (13.09)	85,289 (20.09)
Intermediate	258,021 (60.00)	3150 (57.34)	254,871 (60.03)
High	86,007 (20.00)	1625 (29.58)	84,382 (19.88)

*Note*: The GI‐PRS was categorized into low (bottom quintile), intermediate (quintiles 2–4) and high (top quintile) genetic risk.

Abbreviations: DBP, diastolic blood pressure; HbA1c, glycated hemoglobin; HDL, high density lipoproteins; GI‐PRS, polygenic risk score of gastrointestinal cancer; MetS, metabolic syndrome; SBP, systolic blood pressure; SD, standard deviation.

**TABLE 2:** Association of metabolic syndrome and its components with risk of gastrointestinal cancer

MetS	*N* Cases/Person‐years	Model 1^a^		Model 2^b^	
		HR (95% CI)	*p* value	HR (95% CI)	*p* value
MetS					
No	3570/3 377 750	Reference		Reference	
Yes	1924/1 145 444	**1.31 (1.23, 1.38)**	<0.0001****	**1.28 (1.21, 1.35)**	<0.0001****
Number of MetS components					
0	476/707 374	Reference		Reference	
1–2	3094/2 670 376	**1.13 (1.02, 1.24)**	0.0176*	**1.12 (1.01, 1.23)**	0.0273*
≥3	1924/1 145 444	**1.45 (1.31, 1.61)**	<0.0001****	**1.41 (1.27, 1.56)**	<0.0001****
*p* trend			<0.0001****		<0.0001****

Abbreviations: CI, confidence interval; HR, hazard ratio; MetS, metabolic syndrome.

^a^ Model 1: Adjusted for age group, gender, qualification, Townsend Deprivation Index, family history of cancer.

^b^ Model 2: Additionally adjusted for smoking status, alcohol consumption, physical activity, fruit intake, vegetable consumption, red and processed meat consumption and regular aspirin or ibuprofen use.

*p < 0.05.

****p < 0.0001.

Gastrointestinal polygenic risk score showed a normal distribution and incident cases tended to have higher GI‐PRS than non‐cancer participants (Table 1; Figure S6A). Additionally, GI‐PRS was not associated with MetS, but in association with GI cancer risk, with a HR of 1.35 (95% CI: 1.32–1.39, *p* < 0.0001) per SD of GI‐PRS increase (Figure S6B). In multivariable‐adjusted analyses, there was an obvious gradient in GI cancer risk across quintiles of genetic score (Figure 1C). The risk of GI cancer increased 1.46‐fold and 2.28‐fold in those at intermediate (95% CI: 1.35–1.59, *p* < 0.0001) and high (95% CI: 2.09–2.49, *p* < 0.0001, Figure 1D) genetic risk, respectively, compared with participants at low genetic risk. The site‐specific PRSs were independent from each other (ESC‐GC: Correlation coefficient *r* = −0.001; ESC‐CRC: Correlation coefficient *r* = 0.013; GC‐CRC: Correlation coefficient *r* = 0.001). After standardizing the mean of each site‐specific cancer PRS to 1, the adjusted GI‐PRS was consistently associated with GI cancer risk, which was comparable to the original GI‐PRS (Table S12). Similarly, with any of GC, ESC, and CRC as outcomes, cancer risk increased with their respective increasing PRS (Figure S7). Interestingly, participants with high GI‐PRS tended to have high PRS in multiple GI cancers and the risk of GI cancer increased as the number of high‐genetic risk for site‐specific GI cancers increased. (Table S13).

**FIGURE 2:**
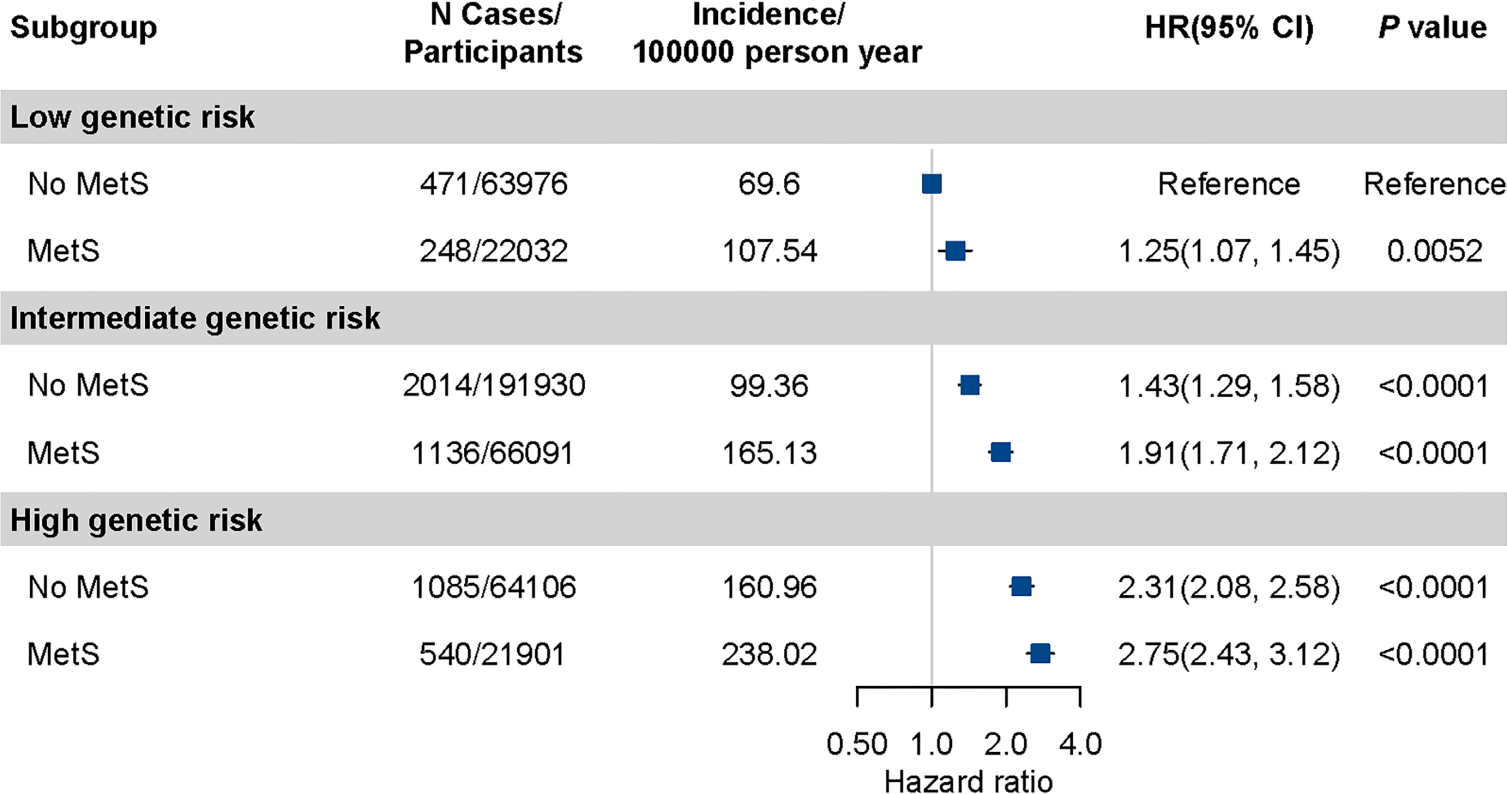
The joint effect of metabolic syndrome and genetic categories on gastrointestinal cancer risk. Models adjusted for age group, gender, qualification, Townsend Deprivation Index, family history of cancer, physical activity, smoking status, alcohol consumption, fruit intake, vegetable consumption, red and processed meat consumption, regular aspirin or ibuprofen use and the top 10 genetic principal components.

When GI‐PRS and MetS were combined, the incidence rate of GI cancer increased with increasing genetic risk and having MetS (Figure 2). Compared with participants in no MetS group and low‐genetic risk, participants having MetS and at high‐genetic risk had an approximately 2.75‐fold increased risk of incident GI cancer (HR = 2.75, 95% CI: 2.43–3.12, *p* < 0.0001). These patterns did not change with restriction to participants in unrelated British ancestry (Figure S8). This dose–response manner across combined subgroups of genetic risk and MetS was also noted for ESC, GC, and CRC (Figure S9).

In the further stratification analyses according to genetic risk, MetS contributed to 34% (95% CI: 1.24–1.45, *p* < 0.0001) and 19% (95% CI: 1.07–1.33, *p* = 0.0012) higher risk, and resulted in 2.75‰ and 3.37‰ absolute risk increases in 5 years among participants at intermediate‐ and high‐genetic risk, in comparison to no MetS with 1.49‰ absolute risk increase, respectively (Table 3). These risk elevations translated into the number of subjects diagnosed as MetS related GI cancer cases in 5 years were 669, 364, and 296 for three risk groups, respectively. Similar patterns were noted in analyses for ESC, GC, and CRC (Table S14). The genetic factor and MetS had no significant interaction on the multiplicative scale (*p*_interaction_ = 0.32), indicating that associations of genetic factor and MetS with GI cancer were independent of each other.

**TABLE 3:** Risk of gastrointestinal cancer according to metabolic syndrome within each genetic risk category

MetS category	Low genetic risk			Intermediate genetic risk			High genetic risk	
	No MetS	MetS		No MetS	MetS		No MetS	MetS
No. of cases/Person years	471/453 059	248/155 700		2014/1 358 078	1136/465 412		1085/452 626	540/153 981
Hazard ratio (95% CI)	Ref.	**1.21 (1.03, 1.42)**		Ref.	**1.34 (1.24, 1.45)**		Ref.	**1.19 (1.07, 1.33)**
*P* value		0.0193*			<0.0001****			0.0012**
Absolute risk (‰)—5 years (95% CI)	2.73 (2.37, 3.09)	4.22 (3.56, 4.88)		4.13 (3.87, 4.38)	6.88 (6.38, 7.38)		7.01 (6.43, 7.59)	10.39 (9.32, 11.45)
Absolute risk increase (‰)—5 years (95% CI)		1.49 (0.85, 2.10)			2.75 (2.30, 3.18)			3.37 (2.31, 4.41)
Numbers—5 years^a^ (95% CI)		669 (213, 871)			364 (296, 415)			296 (166, 367)

*Note*: Models adjusted for age group, gender, qualification, Townsend Deprivation Index, family history of cancer, smoking status, alcohol consumption, physical activity, fruit intake, vegetable consumption, red and processed meat consumption, regular aspirin or ibuprofen use and the top 10 genetic principal components.

Abbreviations: CI, confidence interval; HR, hazard ratio; MetS, metabolic syndrome.

^a^ The number of subjects with metabolic syndrome cause a new gastrointestinal cancer case in 5 years.

*p < 0.05.

**p < 0.01.

****p < 0.0001.

## 4 DISCUSSION

In this large‐scale cohort study, we observed that GI cancer risk was higher among participants with MetS than among those without MetS and increased as the number of unfavorable MetS components increased. We generated a composite GI‐PRS to evaluate the genetic risk of overall GI cancer based on PRSs of ESC, GC, and CRC. High GI‐PRS was in association with an elevated risk of incident GI cancer independent from MetS. A high‐genetic risk together with MetS was related to a 2.75‐fold increase in cancer risk compared with a low‐genetic risk and no MetS profile. Importantly, we showed a higher absolute GI cancer risk increase for subjects with versus without MetS in higher genetic risk group. Collectively, these findings further support population‐based efforts to prevent MetS and subsequent GI cancer risk, particularly for individuals with high‐genetic risk.

Unhealthy lifestyle factors could contribute to higher cancer incidence, while adherence to a healthy lifestyle would lower cancer risk.^29^, ^30^ However, lifestyle factors are too complex to measure precisely. MetS as a surrogate marker for unhealthy lifestyle factors can be obtained through anthropometric data and has been associated with many common cancers.^3^, ^9^ In this study, we also confirmed that MetS was a strong predictor of overall GI cancer risk. Of the five MetS components, central obesity and hyperglycemia were independently related to overall GI cancer risk, which was consistent with the fact that these two factors are thought to predominantly contribute to the association between MetS and cancer.^3^ A healthy lifestyle is critical for MetS prevention and management regarding metabolic status,^31^ which may ultimately result in decreased cancer risk. Therefore, MetS as a measurable body indicator may partially reflect lifestyle behaviors to indicate cancer risk.

Polygenic risk score has been used to measure the cumulative genetic burden for many site‐specific cancers.^17^, ^32^, ^33^, ^34^ Meanwhile, more efforts focus on its predictive power for broad diseases. Ganna et al. in 2013 created a genetic score for 125 diseases or risk factors to assess overall mortality risk.^35^ However, this PRS was unweighted by simply adding the amount of risk alleles across 707 trait‐related SNPs. Recently, a study evaluated the utility of a composite PRS constructed by weighted combination of trait‐specific PRSs for 13 diseases and 12 risk factors in association with all‐cause mortality.^36^ More recently, our group constructed a CPRS to effectively indicate genetic risk for overall cancer.^13^ As we know, this study is the first to evaluate the feasibility of GI‐PRS for genetic risk prediction of overall GI cancer. We confirmed that GI‐PRS was in robust association with incident GI cancer events, which is aligned with the trend for site‐specific PRSs in our analyses and previous studies.^10^, ^17^ That means that GI‐PRS could be utilized as a measurable indicator to identify populations at high‐genetic risk of GI cancer.

Previous studies have indicated that a fraction of incident cancers could be prevented if eliminating or reducing exposure to modifiable risk factors,^37^, ^38^ and a healthy lifestyle may counteract a high‐genetic risk of cancer.^10^, ^13^ In our study, we found a joint effect between GI‐PRS and MetS, suggesting that individuals genetically predisposed to GI cancer may benefit more from MetS prevention. Additionally, MetS was independent from GI‐PRS on GI cancer risk, which was inconsistent with that of healthy lifestyle.^10^ Therefore, our findings provided the first evidence that MetS could act as an *in vivo* surrogate indicator to assess the effect of GI cancer risk reduction from healthy lifestyles and metabolic status, especially for high‐genetic risk populations. Since genetic risk is unchangeable, lifestyle or medical interventions targeting at populations with MetS and high‐genetic risk would be effective precautions for GI cancer prevention.

The major advantages of our study are the large sample size based on a prospective cohort, together with comprehensive and detailed information about anthropometry. Furthermore, we developed a novel marker to predict the genetic risk of overall GI cancer, and combined it with MetS to explore the conjoint effect between genetic and metabolic risk factors for the first time. Yet, our study has several limitations. First, MetS components were measured at baseline, and behavioral changes or medical intervention during follow‐up might change MetS status and bias the risk estimates. Second, since about three‐quarters of incident GI cancer cases were CRC, the results should be interpreted with caution for ESC and GC although three cancers showed similar association patterns. Third, different numbers of genetic loci were reported for three cancers, which may result in imbalanced weights of different cancers to GI‐PRS. Finally, since our analyses were performed restricted in white population, this limits the extrapolation of our findings to individuals of other ethnic backgrounds.

In conclusion, the findings of this study suggest that the measurable MetS as a surrogate indicator of unhealthy lifestyles may be a risk predictor for overall GI cancer, while the newly constructed GI‐PRS can act as a measurable genetic indicator. Metabolic and genetic factors may jointly contribute to GI cancer risk. MetS and GI‐PRS may serve as predictors of GI cancer risk to identify high‐risk populations for precise prevention.

## AUTHOR CONTRIBUTIONS

**Meng Zhu, Li Liu** and **Guangfu Jin:** Conceptualization. **Yaqian Liu** and **Caiwang Yan**: Methodology; **Yaqian Liu** and **Caiwang Yan:** Formal analysis and investigation; **Yaqian Liu:** Writing – original draft preparation. **Yaqian Liu** and **Caiwang Yan:** Writing – review & editing: **Yaqian Liu, Caiwang Yan, Shuangshuang Yin, Tianpei Wang, Meng Zhu, Li Liu** and **Guangfu Jin:** Data acquisition or interpretation. All authors have read and approved the publication of the final manuscript.

## CONFLICTS OF INTEREST

The authors disclose no conflicts.

## Supporting information


Figure S1

Figure S2

Figure S3

Figure S4

Figure S5

Figure S6

Figure S7

Figure S8

Figure S9

Table S1
Table S2
Table S3
Table S4
Table S5
Table S6
Table S7
Table S8
Table S9
Table S10
Table S11
Table S12
Table S13
Table S14

## Data Availability

Information about data access is available at https://biobank.ndph.ox.ac.uk/ukb/.
