# Supplementary figures and images for: Genetic risk, metabolic syndrome, and gastrointestinal cancer risk: A prospective cohort study
Source: Cancer Med. 2022 Jun 22;12(1):597–605. doi: 10.1002/cam4.4923 (PMC9844643; doi:10.1002/cam4.4923)

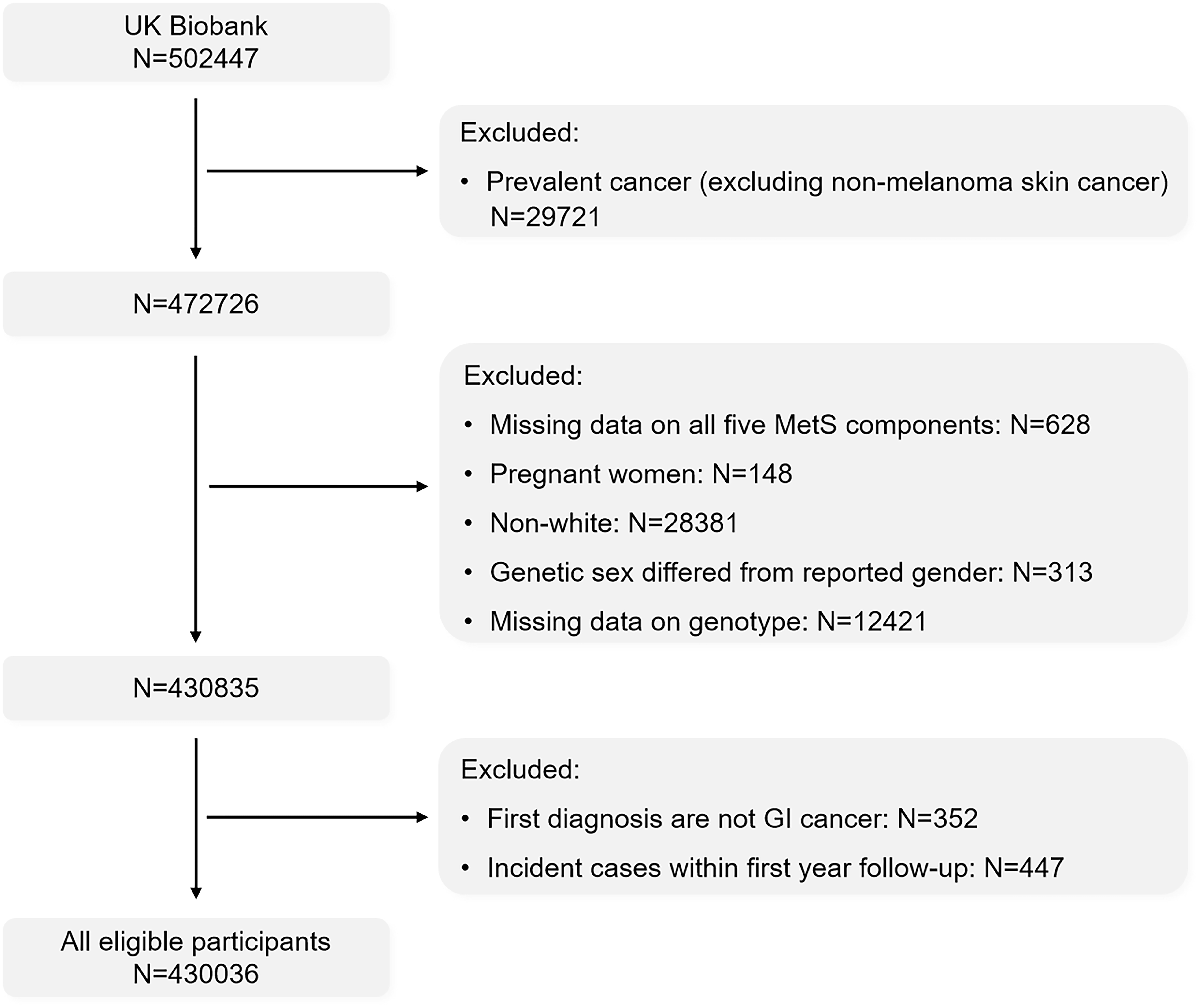

Supplement: Supplementary file 1 — Figure S1 [file CAM4-12-597-s001.tif]

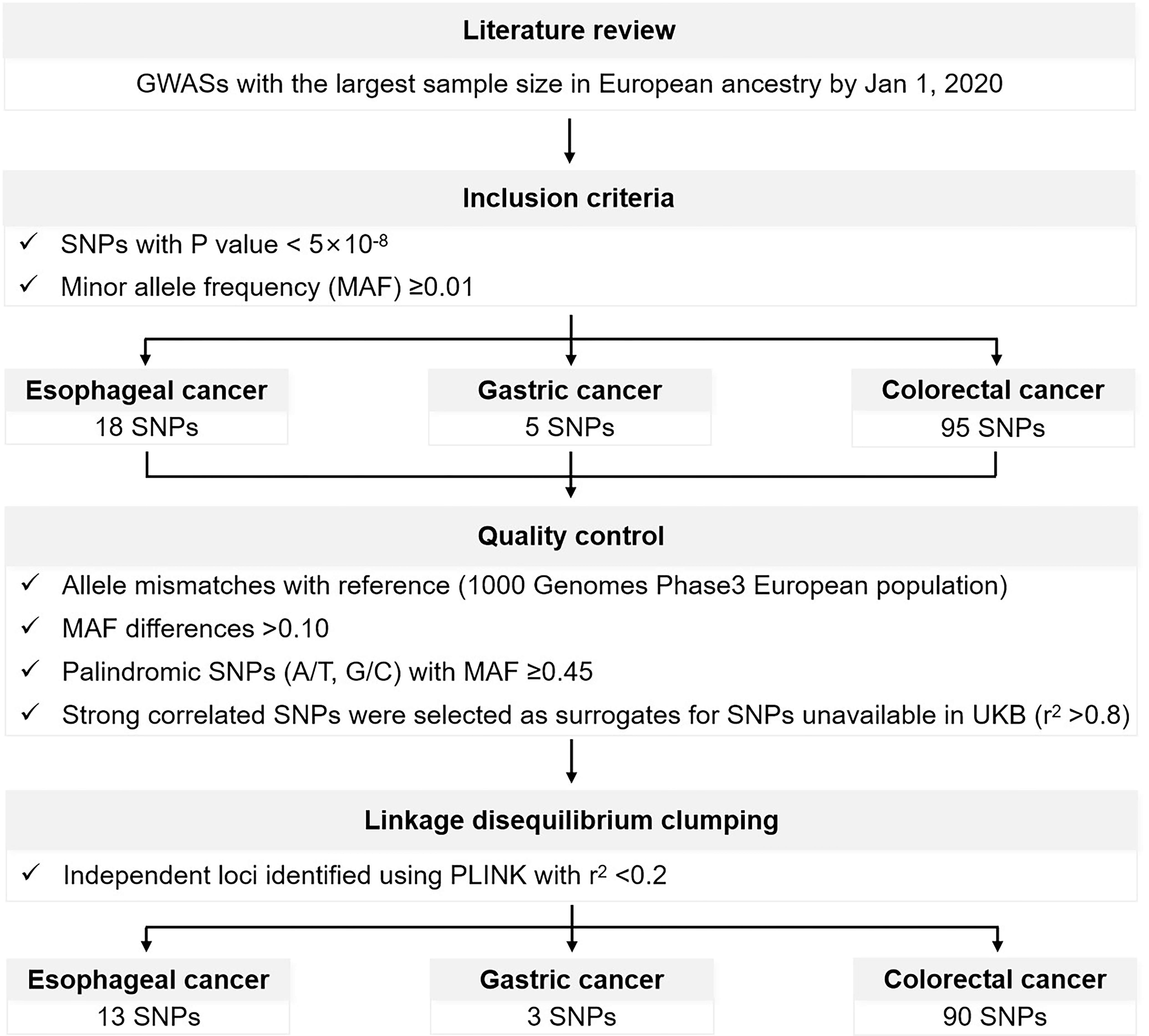

Supplement: Supplementary file 2 — Figure S2 [file CAM4-12-597-s002.jpg]

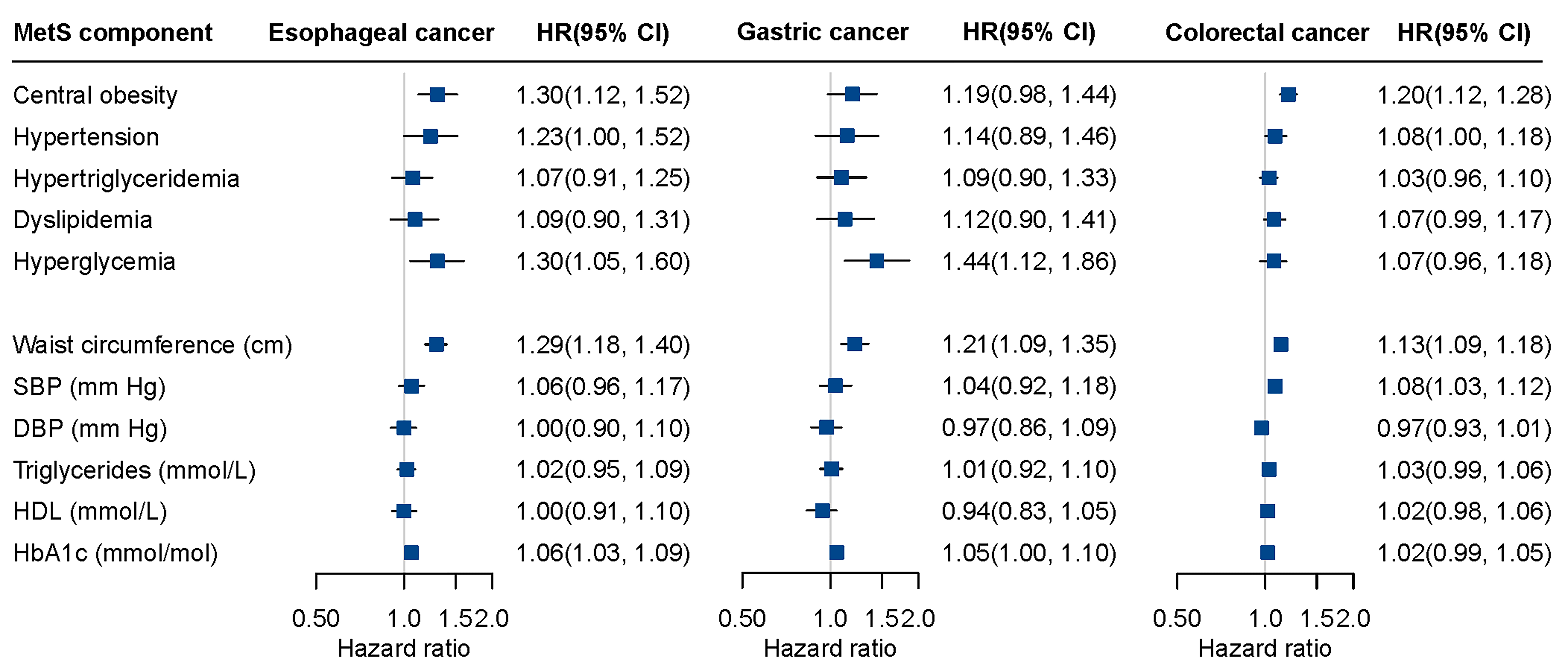

Supplement: Supplementary file 3 — Figure S3 [file CAM4-12-597-s008.tiff]

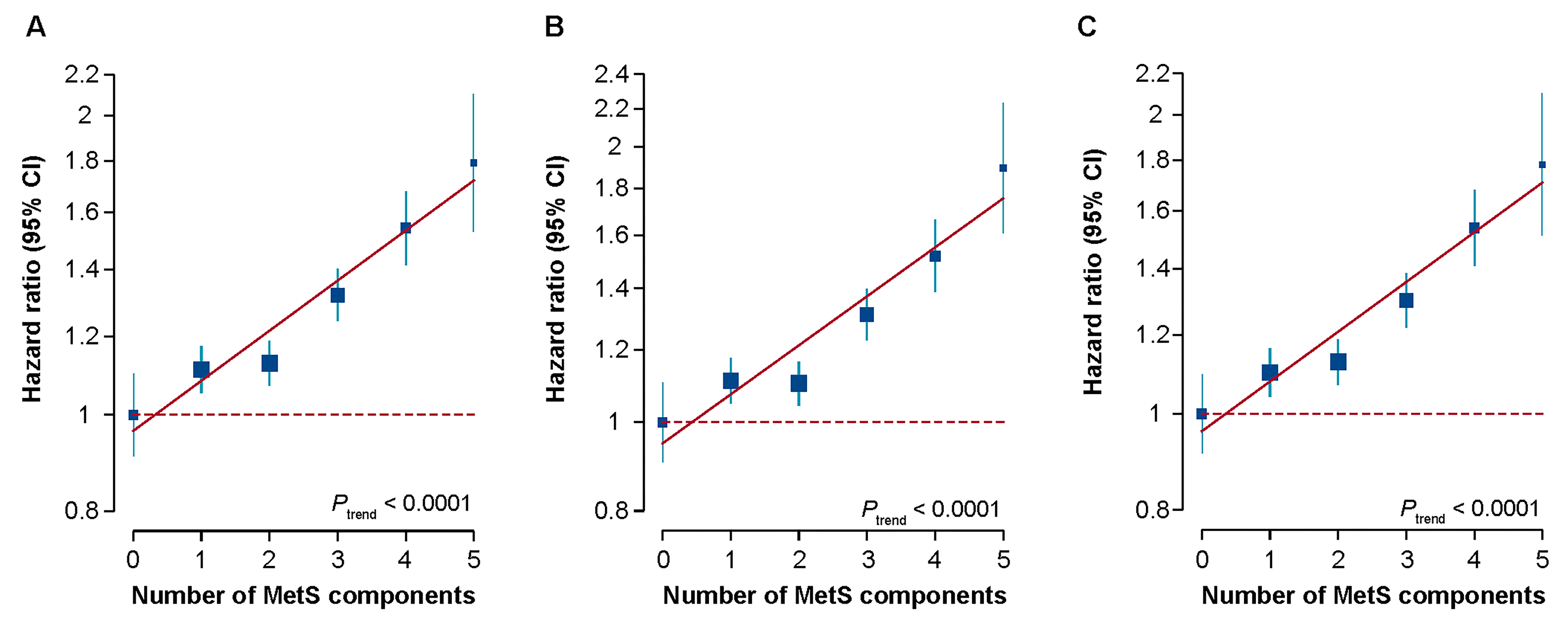

Supplement: Supplementary file 4 — Figure S4 [file CAM4-12-597-s003.tiff]

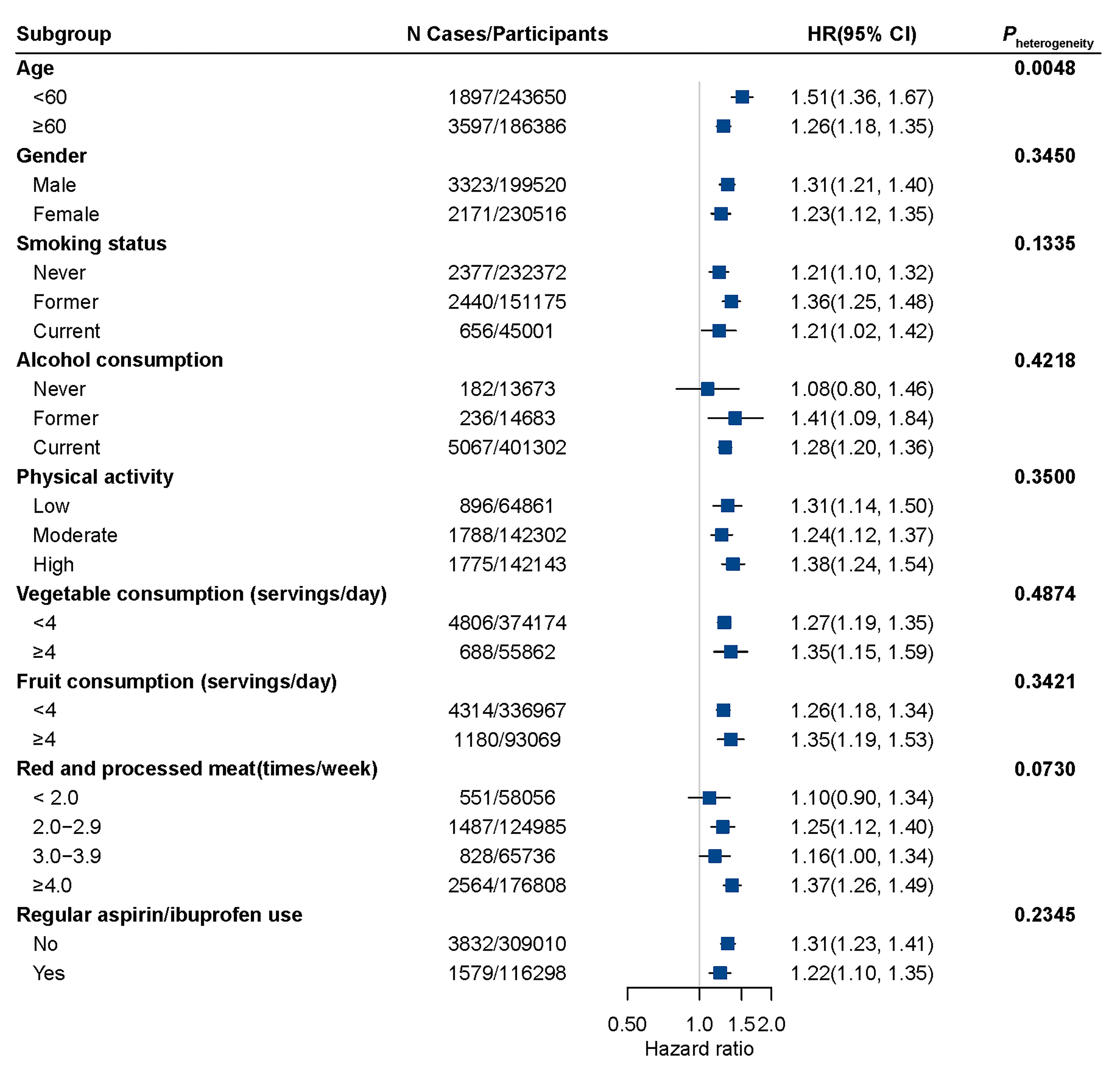

Supplement: Supplementary file 5 — Figure S5 [file CAM4-12-597-s005.tiff]

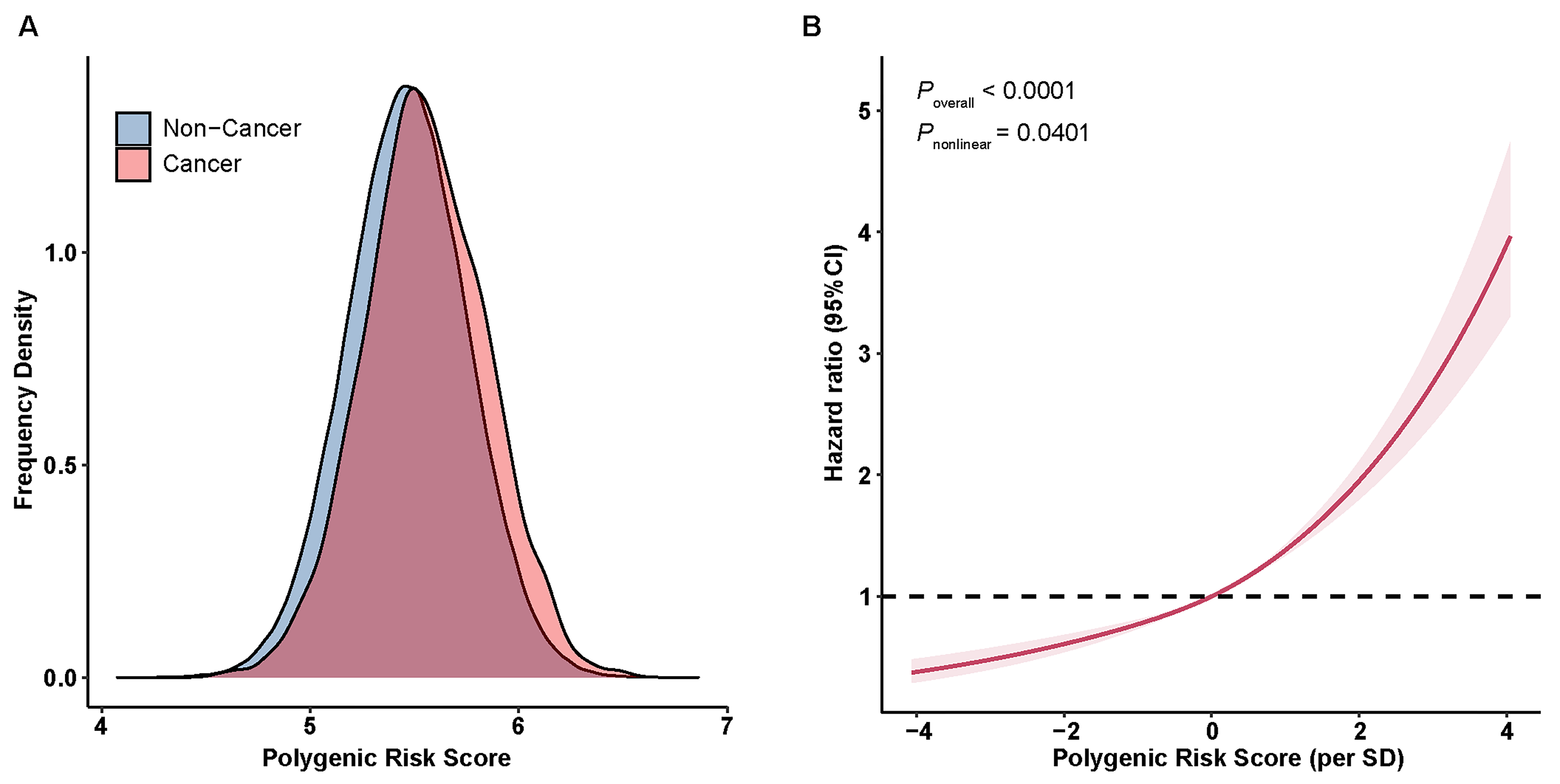

Supplement: Supplementary file 6 — Figure S6 [file CAM4-12-597-s007.tiff]

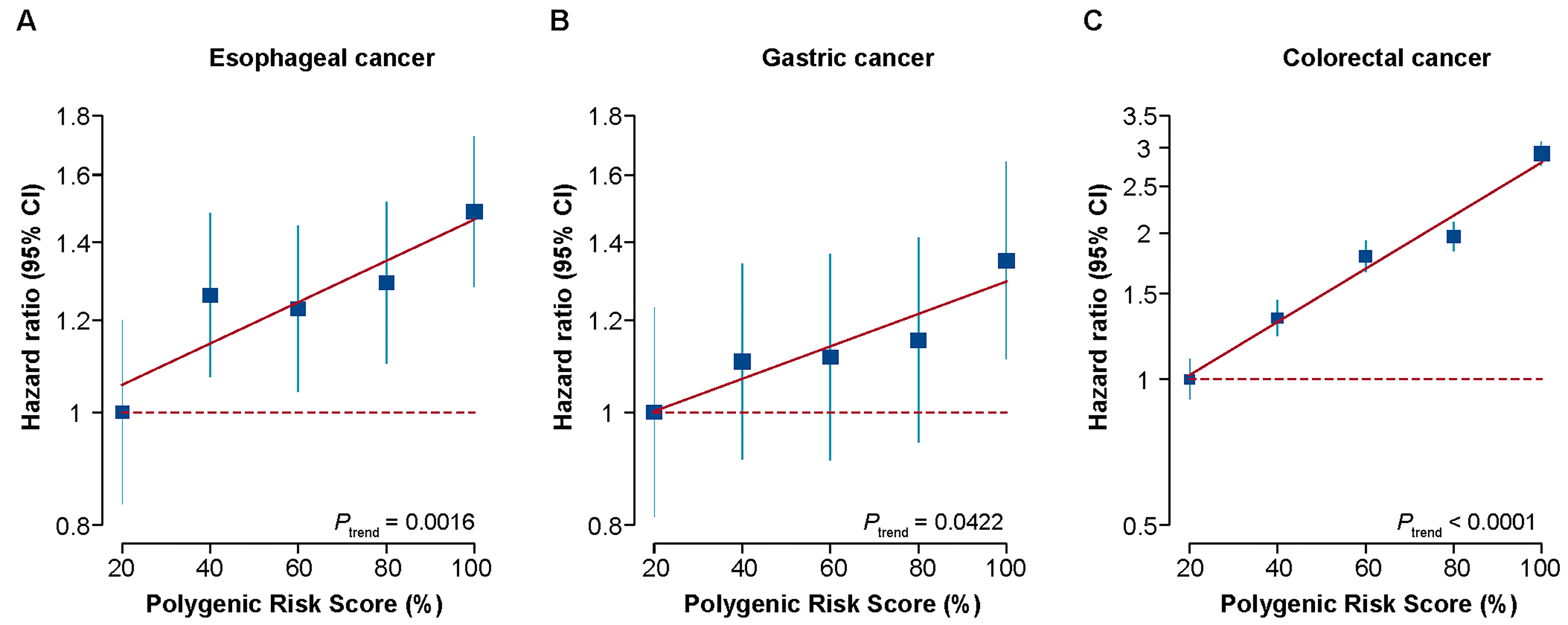

Supplement: Supplementary file 7 — Figure S7 [file CAM4-12-597-s010.tiff]

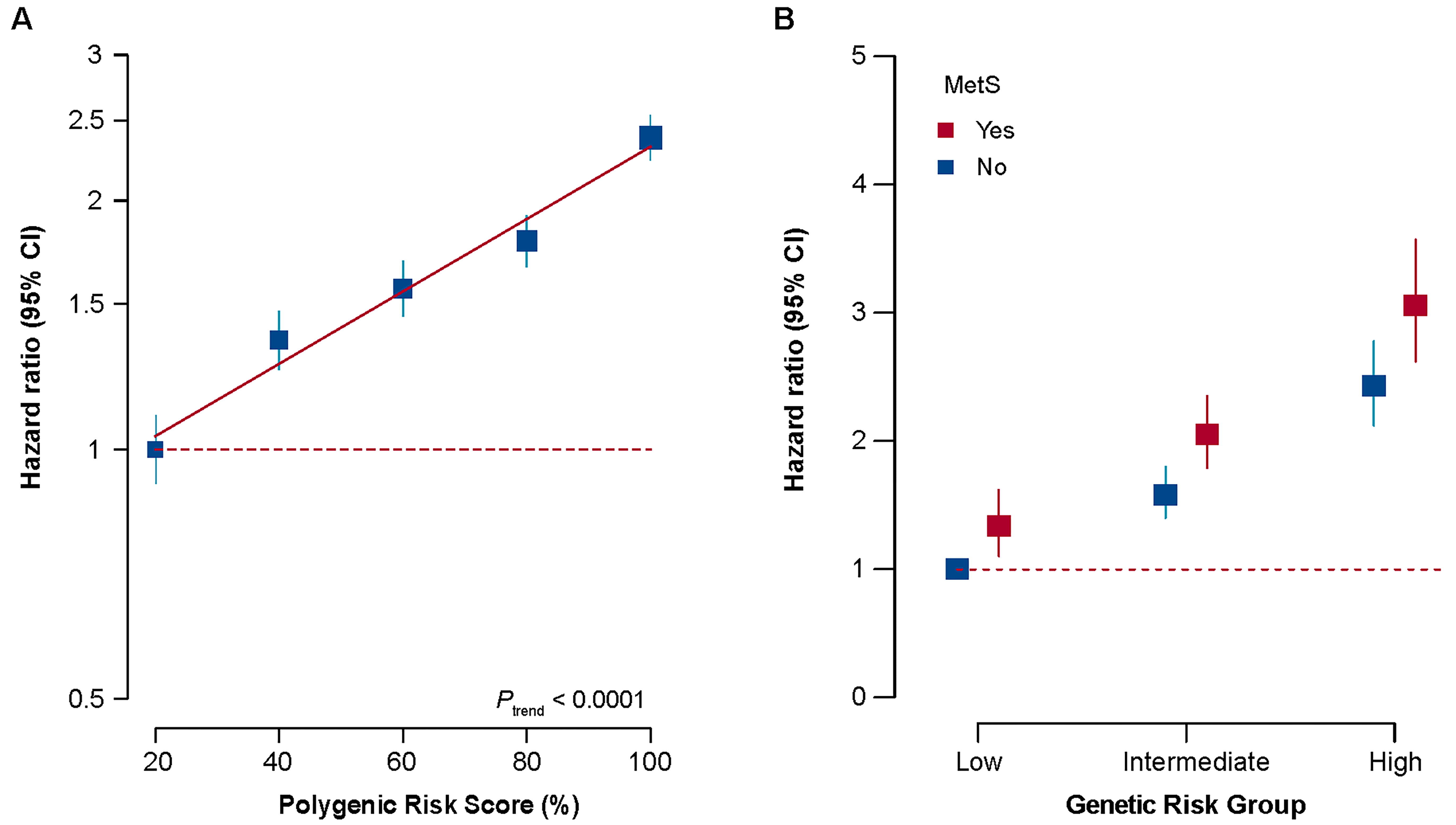

Supplement: Supplementary file 8 — Figure S8 [file CAM4-12-597-s004.tiff]

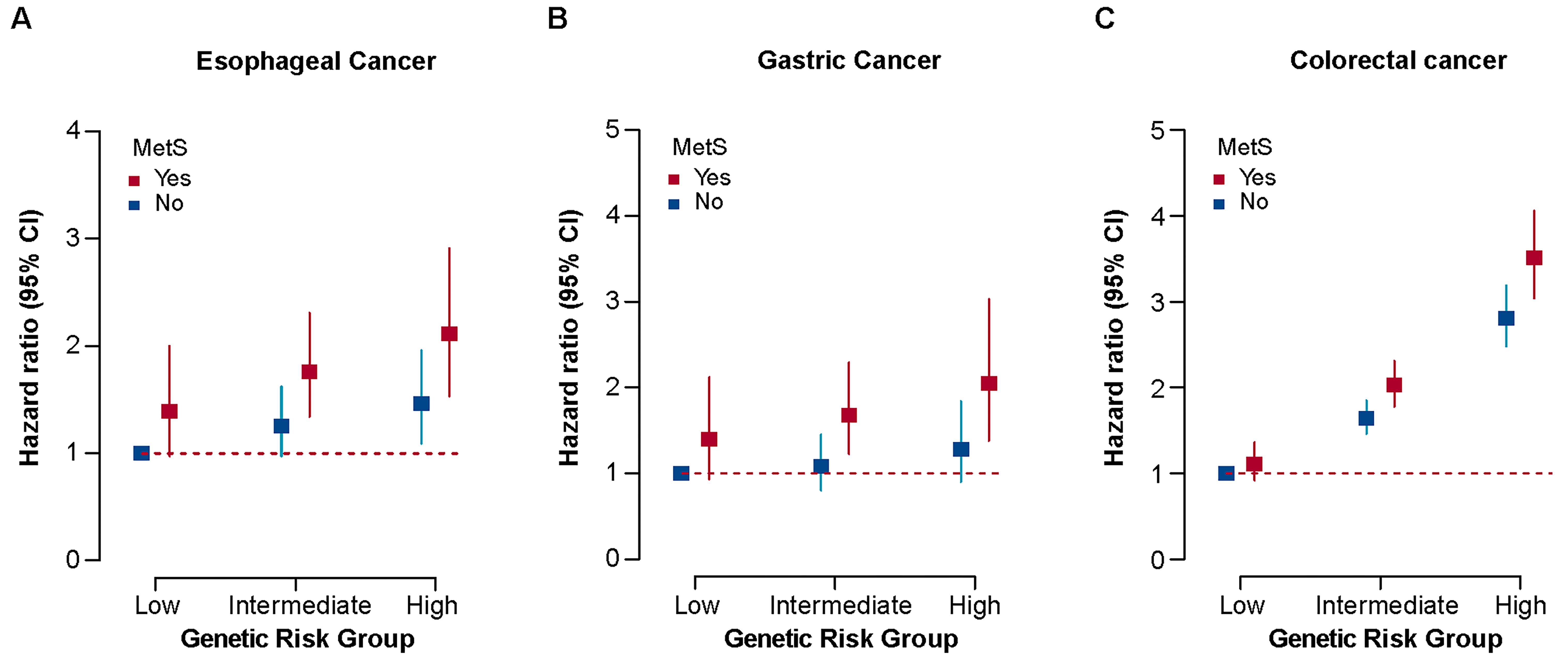

Supplement: Supplementary file 9 — Figure S9 [file CAM4-12-597-s009.tiff]
