# Supplementary material for: Genetic risk, metabolic syndrome, and gastrointestinal cancer risk: A prospective cohort study
Source: Cancer Med. 2022 Jun 22;12(1):597–605. doi: 10.1002/cam4.4923 (PMC9844643; doi:10.1002/cam4.4923)
Supplement: Supplementary file 10 — Table S1 Table S2 Table S3 Table S4 Table S5 Table S6 Table S7 Table S8 Table S9 Table S10 Table S11 Table S12 Table S13 Table S14 [file CAM4-12-597-s006.docx]

**Table S1. Definition of metabolic syndrome according to NCEP-ATP III**

| **MetS component** | **Definition** |
| --- | --- |
| Central obesity | waist circumference ≥102 cm for men or ≥88 cm for women |
| Hypertension | systolic blood pressure ≥130 mmHg and/or diastolic blood pressure ≥85 mmHg or on therapy with antihypertensive medication |
| Hypertriglyceridemia | HDL cholesterol level <1 mmol/L for men or <1.3 mmol/L for women |
| Dyslipidemia | triglycerides level ≥1.7 mmol/L or taking triglyceride-lowering drugs |
| Hyperglycemia | fasting blood glucose level ≥6.1 mmol/L or taking hypoglycemic medication |

HDL, high density lipoproteins.

**Table S2.** Single nucleotide polymorphisms utilized to build the polygenic risk scores for site-specific gastrointestinal cancers

| Index | ICD10-code | Cancer site | ASR^a^ | No. of SNPs included | SNP rsID^b^ | Chr. | Position (GRCh37) | Risk  Allele | Other  Allele | Weight | Study (PMID) | Comment |
| --- | --- | --- | --- | --- | --- | --- | --- | --- | --- | --- | --- | --- |
| 1 | C15 | Oesophagus | 14.7 | 13 | rs3072 | 2 | 20878406 | C | T | 0.131028262 | 30243622 | - |
| 2 | C15 | Oesophagus | 14.7 | 13 | rs7255 | 2 | 20878820 | T | C | 0.157003749 | 30243622 | Removed after LD clumping |
| 3 | C15 | Oesophagus | 14.7 | 13 | rs13397172 | 2 | 200045039 | C | T | 0.122217633 | 30243622 | - |
| 4 | C15 | Oesophagus | 14.7 | 13 | rs2687202 | 3 | 70929983 | T | C | 0.122217633 | 30243622 | - |
| 5 | C15 | Oesophagus | 14.7 | 13 | rs9823696 | 3 | 183783353 | A | G | 0.157003749 | 30243622 | - |
| 6 | C15 | Oesophagus | 14.7 | 13 | rs62423175 | 6 | 62195368 | A | G | 0.207014169 | 30243622 | - |
| 7 | C15 | Oesophagus | 14.7 | 13 | rs2188554 | 7 | 117040117 | A | G | 0.207014169 | 30243622 | Removed after LD clumping |
| 8 | C15 | Oesophagus | 14.7 | 13 | rs17451754 | 7 | 117256712 | G | A | 0.223143551 | 30243622 | - |
| 9 | C15 | Oesophagus | 14.7 | 13 | rs17749155 | 8 | 10068073 | A | G | 0.131028262 | 30243622 | - |
| 10 | C15 | Oesophagus | 14.7 | 13 | rs10108511 | 8 | 11435516 | T | C | 0.113328685 | 30243622 | - |
| 11 | C15 | Oesophagus | 14.7 | 13 | rs11789015 | 9 | 96716028 | A | G | 0.182321557 | 30243622 | - |
| 12 | C15 | Oesophagus | 14.7 | 13 | rs7852462 | 9 | 100310501 | C | T | 0.076961041 | 30243622 | - |
| 13 | C15 | Oesophagus | 14.7 | 13 | rs1247942 | 12 | 114673723 | G | C | 0.104360015 | 30243622 | unspecified allele |
| 14 | C15 | Oesophagus | 14.7 | 13 | rs66725070 | 15 | 58267416 | GACAT | - | 0.139761942 | 30243622 | Unavailable in UK Biobank |
| 15 | C15 | Oesophagus | 14.7 | 13 | rs2464469 | 15 | 58362025 | G | A | 0.104360015 | 30243622 | - |
| 16 | C15 | Oesophagus | 14.7 | 13 | rs1979654 | 16 | 86396835 | G | C | 0.104360015 | 30243622 | unspecified allele |
| 17 | C15 | Oesophagus | 14.7 | 13 | rs10419226 | 19 | 18803172 | T | G | 0.165514438 | 30243622 | - |
| 18 | C15 | Oesophagus | 14.7 | 13 | rs10423674 | 19 | 18817903 | C | A | 0.173953307 | 30243622 | - |
| 19 | C16 | Stomach | 9.9 | 3 | rs140081212 | 1 | 153451599 | G | A | 0.235722334 | 26098866 | Unavailable in UK Biobank |
| 20 | C16 | Stomach | 9.9 | 3 | rs760077 | 1 | 153445406 | T | A | 0.235722334 | 26098866 | - |
| 21 | C16 | Stomach | 9.9 | 3 | rs6676150 | 1 | 153390461 | G | C | 0.235722334 | 26098866 | Removed after LD clumping |
| 22 | C16 | Stomach | 9.9 | 3 | rs2920295 | 8 | 143764937 | G | A | 0.19062036 | 26098866 | - |
| 23 | C16 | Stomach | 9.9 | 3 | rs10036575 | 5 | 40685795 | T | C | 0.210721031 | 26098866 | - |
| 24 | C18-C20 | Colon, Rectum | 67.3 | 90 | rs72647484 | 1 | 22587728 | T | C | 0.0504 | 30510241 | - |
| 25 | C18-C20 | Colon, Rectum | 67.3 | 90 | rs4360494 | 1 | 38455891 | G | C | 0.0523 | 30510241 | unspecified allele |
| 26 | C18-C20 | Colon, Rectum | 67.3 | 90 | rs12144319 | 1 | 55246035 | C | T | 0.0665 | 30510241 | - |
| 27 | C18-C20 | Colon, Rectum | 67.3 | 90 | rs6678517 | 1 | 183002639 | A | G | 0.073 | 30510241 | - |
| 28 | C18-C20 | Colon, Rectum | 67.3 | 90 | rs17011141 | 1 | 222112634 | G | A | 0.0877 | 30510241 | - |
| 29 | C18-C20 | Colon, Rectum | 67.3 | 90 | rs448513 | 2 | 159964552 | C | T | 0.0511 | 30510241 | - |
| 30 | C18-C20 | Colon, Rectum | 67.3 | 90 | rs11884596 | 2 | 199612407 | C | T | 0.0535 | 30510241 | - |
| 31 | C18-C20 | Colon, Rectum | 67.3 | 90 | rs983402 | 2 | 199781586 | T | C | 0.0627 | 30510241 | - |
| 32 | C18-C20 | Colon, Rectum | 67.3 | 90 | rs3731861 | 2 | 219191256 | T | C | 0.0613 | 30510241 | - |
| 33 | C18-C20 | Colon, Rectum | 67.3 | 90 | rs35470271 | 3 | 40915239 | G | A | 0.0994 | 30510241 | - |
| 34 | C18-C20 | Colon, Rectum | 67.3 | 90 | rs6781752 | 3 | 66365163 | A | G | 0.0597 | 30510241 | - |
| 35 | C18-C20 | Colon, Rectum | 67.3 | 90 | rs72942485 | 3 | 112999560 | G | A | 0.1761 | 30510241 | - |
| 36 | C18-C20 | Colon, Rectum | 67.3 | 90 | rs10049390 | 3 | 133701119 | A | G | 0.0597 | 30510241 | - |
| 37 | C18-C20 | Colon, Rectum | 67.3 | 90 | rs9876206 | 3 | 169517436 | C | T | 0.0453 | 30510241 | - |
| 38 | C18-C20 | Colon, Rectum | 67.3 | 90 | rs13149359 | 4 | 94938618 | A | C | 0.052 | 30510241 | - |
| 39 | C18-C20 | Colon, Rectum | 67.3 | 90 | rs1391441 | 4 | 106128760 | A | G | 0.0522 | 30510241 | - |
| 40 | C18-C20 | Colon, Rectum | 67.3 | 90 | rs11727676 | 4 | 145659064 | C | T | 0.0842 | 30510241 | - |
| 41 | C18-C20 | Colon, Rectum | 67.3 | 90 | rs78368589 | 5 | 1240204 | T | C | 0.1119 | 30510241 | - |
| 42 | C18-C20 | Colon, Rectum | 67.3 | 90 | rs2735940 | 5 | 1296486 | G | A | 0.0865 | 30510241 | - |
| 43 | C18-C20 | Colon, Rectum | 67.3 | 90 | rs7708610 | 5 | 40102443 | A | G | 0.0545 | 30510241 | - |
| 44 | C18-C20 | Colon, Rectum | 67.3 | 90 | rs12514517 | 5 | 40280076 | A | G | 0.1013 | 30510241 | - |
| 45 | C18-C20 | Colon, Rectum | 67.3 | 90 | rs145364999 | 5 | 98206082 | T | A | 0.5559 | 30510241 | - |
| 46 | C18-C20 | Colon, Rectum | 67.3 | 90 | rs755229494 | 5 | 112097351 | G | A | 0.6286 | 30510241 | - |
| 47 | C18-C20 | Colon, Rectum | 67.3 | 90 | rs4976270 | 5 | 134467220 | C | T | 0.0693 | 30510241 | - |
| 48 | C18-C20 | Colon, Rectum | 67.3 | 90 | rs2516420 | 6 | 31449620 | C | T | 0.1118 | 30510241 | - |
| 49 | C18-C20 | Colon, Rectum | 67.3 | 90 | rs9271695 | 6 | 32593080 | G | A | 0.0889 | 30510241 | - |
| 50 | C18-C20 | Colon, Rectum | 67.3 | 90 | rs16878812 | 6 | 35569562 | A | G | 0.0778 | 30510241 | - |
| 51 | C18-C20 | Colon, Rectum | 67.3 | 90 | rs9470361 | 6 | 36623379 | A | G | 0.054 | 30510241 | - |
| 52 | C18-C20 | Colon, Rectum | 67.3 | 90 | rs62396735 | 6 | 41702582 | C | T | 0.033 | 30510241 | - |
| 53 | C18-C20 | Colon, Rectum | 67.3 | 90 | rs62404966 | 6 | 55712124 | C | T | 0.0724 | 30510241 | - |
| 54 | C18-C20 | Colon, Rectum | 67.3 | 90 | rs12672022 | 7 | 45136423 | T | C | 0.065 | 30510241 | - |
| 55 | C18-C20 | Colon, Rectum | 67.3 | 90 | rs16892766 | 8 | 117630683 | C | A | 0.2099 | 30510241 | Removed after LD clumping |
| 56 | C18-C20 | Colon, Rectum | 67.3 | 90 | rs6469654 | 8 | 117632965 | G | C | 0.0677 | 30510241 | - |
| 57 | C18-C20 | Colon, Rectum | 67.3 | 90 | rs117079142 | 8 | 117790914 | A | C | 0.1139 | 30510241 | - |
| 58 | C18-C20 | Colon, Rectum | 67.3 | 90 | rs6983267 | 8 | 128413305 | G | T | 0.1052 | 30510241 | - |
| 59 | C18-C20 | Colon, Rectum | 67.3 | 90 | rs4313119 | 8 | 128571855 | G | T | 0.0608 | 30510241 | - |
| 60 | C18-C20 | Colon, Rectum | 67.3 | 90 | rs1537372 | 9 | 22103183 | G | T | 0.0504 | 30510241 | - |
| 61 | C18-C20 | Colon, Rectum | 67.3 | 90 | rs34405347 | 9 | 101679752 | T | G | 0.0818 | 30510241 | - |
| 62 | C18-C20 | Colon, Rectum | 67.3 | 90 | rs10980628 | 9 | 113671403 | C | T | 0.0637 | 30510241 | - |
| 63 | C18-C20 | Colon, Rectum | 67.3 | 90 | rs11255841 | 10 | 8739580 | T | A | 0.1064 | 30510241 | - |
| 64 | C18-C20 | Colon, Rectum | 67.3 | 90 | rs10821907 | 10 | 52648454 | C | T | 0.073 | 30510241 | - |
| 65 | C18-C20 | Colon, Rectum | 67.3 | 90 | rs704017 | 10 | 80819132 | G | A | 0.0765 | 30510241 | - |
| 66 | C18-C20 | Colon, Rectum | 67.3 | 90 | rs11190164 | 10 | 101351704 | G | A | 0.0889 | 30510241 | - |
| 67 | C18-C20 | Colon, Rectum | 67.3 | 90 | rs12246635 | 10 | 114288619 | C | T | 0.0975 | 30510241 | - |
| 68 | C18-C20 | Colon, Rectum | 67.3 | 90 | rs11196170 | 10 | 114722621 | A | G | 0.0527 | 30510241 | - |
| 69 | C18-C20 | Colon, Rectum | 67.3 | 90 | rs174533 | 11 | 61549025 | G | A | 0.0636 | 30510241 | - |
| 70 | C18-C20 | Colon, Rectum | 67.3 | 90 | rs7121958 | 11 | 74280012 | G | T | 0.078 | 30510241 | - |
| 71 | C18-C20 | Colon, Rectum | 67.3 | 90 | rs61389091 | 11 | 74427921 | C | T | 0.1934 | 30510241 | - |
| 72 | C18-C20 | Colon, Rectum | 67.3 | 90 | rs2186607 | 11 | 101656397 | T | A | 0.0537 | 30510241 | unspecified allele |
| 73 | C18-C20 | Colon, Rectum | 67.3 | 90 | rs3087967 | 11 | 111156836 | T | C | 0.1122 | 30510241 | - |
| 74 | C18-C20 | Colon, Rectum | 67.3 | 90 | rs35808169 | 12 | 4368607 | C | T | 0.089 | 30510241 | - |
| 75 | C18-C20 | Colon, Rectum | 67.3 | 90 | rs3217810 | 12 | 4388271 | T | C | 0.1181 | 30510241 | - |
| 76 | C18-C20 | Colon, Rectum | 67.3 | 90 | rs3217874 | 12 | 4400808 | T | C | 0.055 | 30510241 | - |
| 77 | C18-C20 | Colon, Rectum | 67.3 | 90 | rs2250430 | 12 | 6421174 | T | A | 0.0597 | 30510241 | - |
| 78 | C18-C20 | Colon, Rectum | 67.3 | 90 | rs2710310 | 12 | 12035649 | C | T | 0.0145 | 30510241 | - |
| 79 | C18-C20 | Colon, Rectum | 67.3 | 90 | rs11610543 | 12 | 43134191 | G | A | 0.053 | 30510241 | - |
| 80 | C18-C20 | Colon, Rectum | 67.3 | 90 | rs12372718 | 12 | 51171090 | G | A | 0.0896 | 30510241 | - |
| 81 | C18-C20 | Colon, Rectum | 67.3 | 90 | rs4759277 | 12 | 57533690 | A | C | 0.053 | 30510241 | - |
| 82 | C18-C20 | Colon, Rectum | 67.3 | 90 | rs597808 | 12 | 111973358 | G | A | 0.0737 | 30510241 | - |
| 83 | C18-C20 | Colon, Rectum | 67.3 | 90 | rs7300312 | 12 | 115890922 | C | T | 0.066 | 30510241 | - |
| 84 | C18-C20 | Colon, Rectum | 67.3 | 90 | rs377429877 | 13 | 34092165 | C | T | 0.0468 | 30510241 | Unavailable in UK Biobank |
| 85 | C18-C20 | Colon, Rectum | 67.3 | 90 | rs7333607 | 13 | 37462010 | G | A | 0.0758 | 30510241 | - |
| 86 | C18-C20 | Colon, Rectum | 67.3 | 90 | rs78341008 | 13 | 73791554 | C | T | 0.0982 | 30510241 | - |
| 87 | C18-C20 | Colon, Rectum | 67.3 | 90 | rs8000189 | 13 | 111075881 | T | C | 0.0549 | 30510241 | - |
| 88 | C18-C20 | Colon, Rectum | 67.3 | 90 | rs35107139 | 14 | 54419106 | C | A | 0.0912 | 30510241 | - |
| 89 | C18-C20 | Colon, Rectum | 67.3 | 90 | rs4901473 | 14 | 54445157 | G | A | 0.0465 | 30510241 | - |
| 90 | C18-C20 | Colon, Rectum | 67.3 | 90 | rs17094983 | 14 | 59189361 | G | A | 0.0691 | 30510241 | - |
| 91 | C18-C20 | Colon, Rectum | 67.3 | 90 | rs12708491 | 15 | 32992836 | G | A | 0.0464 | 30510241 | - |
| 92 | C18-C20 | Colon, Rectum | 67.3 | 90 | rs2293581 | 15 | 33010736 | A | G | 0.1248 | 30510241 | - |
| 93 | C18-C20 | Colon, Rectum | 67.3 | 90 | rs17816465 | 15 | 33156386 | A | G | 0.0705 | 30510241 | - |
| 94 | C18-C20 | Colon, Rectum | 67.3 | 90 | rs56324967 | 15 | 67402824 | C | T | 0.0689 | 30510241 | - |
| 95 | C18-C20 | Colon, Rectum | 67.3 | 90 | rs9924886 | 16 | 68743939 | A | C | 0.055 | 30510241 | - |
| 96 | C18-C20 | Colon, Rectum | 67.3 | 90 | rs9930005 | 16 | 80043258 | C | A | 0.0498 | 30510241 | - |
| 97 | C18-C20 | Colon, Rectum | 67.3 | 90 | rs12149163 | 16 | 86339315 | T | C | 0.0487 | 30510241 | - |
| 98 | C18-C20 | Colon, Rectum | 67.3 | 90 | rs62042090 | 16 | 86703949 | T | C | 0.0481 | 30510241 | - |
| 99 | C18-C20 | Colon, Rectum | 67.3 | 90 | rs4968127 | 17 | 809643 | G | A | 0.0514 | 30510241 | - |
| 100 | C18-C20 | Colon, Rectum | 67.3 | 90 | rs1078643 | 17 | 10707241 | A | G | 0.0748 | 30510241 | - |
| 101 | C18-C20 | Colon, Rectum | 67.3 | 90 | rs983318 | 17 | 70413253 | A | G | 0.0595 | 30510241 | - |
| 102 | C18-C20 | Colon, Rectum | 67.3 | 90 | rs75954926 | 17 | 81061048 | G | A | 0.0882 | 30510241 | - |
| 103 | C18-C20 | Colon, Rectum | 67.3 | 90 | rs11874392 | 18 | 46453156 | A | T | 0.1606 | 30510241 | unspecified allele |
| 104 | C18-C20 | Colon, Rectum | 67.3 | 90 | rs34797592 | 19 | 16417198 | T | C | 0.0868 | 30510241 | - |
| 105 | C18-C20 | Colon, Rectum | 67.3 | 90 | rs28840750 | 19 | 33519927 | T | G | 0.1939 | 30510241 | - |
| 106 | C18-C20 | Colon, Rectum | 67.3 | 90 | rs1963413 | 19 | 41871573 | A | G | 0.0441 | 30510241 | - |
| 107 | C18-C20 | Colon, Rectum | 67.3 | 90 | rs73068325 | 19 | 59079096 | T | C | 0.0632 | 30510241 | - |
| 108 | C18-C20 | Colon, Rectum | 67.3 | 90 | rs189583 | 20 | 6376457 | G | C | 0.0795 | 30510241 | - |
| 109 | C18-C20 | Colon, Rectum | 67.3 | 90 | rs994308 | 20 | 6603622 | C | T | 0.0627 | 30510241 | - |
| 110 | C18-C20 | Colon, Rectum | 67.3 | 90 | rs4813802 | 20 | 6699595 | G | T | 0.0819 | 30510241 | - |
| 111 | C18-C20 | Colon, Rectum | 67.3 | 90 | rs28488 | 20 | 6762221 | T | C | 0.0714 | 30510241 | - |
| 112 | C18-C20 | Colon, Rectum | 67.3 | 90 | rs11087784 | 20 | 7740976 | G | A | 0.0874 | 30510241 | - |
| 113 | C18-C20 | Colon, Rectum | 67.3 | 90 | rs6058093 | 20 | 33213196 | C | A | 0.045 | 30510241 | - |
| 114 | C18-C20 | Colon, Rectum | 67.3 | 90 | rs6031311 | 20 | 42666475 | T | C | 0.0597 | 30510241 | - |
| 115 | C18-C20 | Colon, Rectum | 67.3 | 90 | rs6066825 | 20 | 47340117 | A | G | 0.0719 | 30510241 | - |
| 116 | C18-C20 | Colon, Rectum | 67.3 | 90 | rs6063514 | 20 | 49055318 | C | T | 0.0547 | 30510241 | - |
| 117 | C18-C20 | Colon, Rectum | 67.3 | 90 | rs1741640 | 20 | 60932414 | C | T | 0.1146 | 30510241 | - |
| 118 | C18-C20 | Colon, Rectum | 67.3 | 90 | rs2738783 | 20 | 62308612 | T | G | 0.0593 | 30510241 | - |

^a^The age-standardized incidence rates (ASR, per 100,000 person-years) of each cancer in UK was assessed from the Office for National Statistics (https://www.ons.gov.uk/).

^b^Single Nucleotide Polymorphisms (SNPs) were selected from previously published GWASs with the largest sample size for each cancer.

**Table S3. Definition of covariates**

| **Covariates** | **Definition** | **UK Biobank**  **Field code** |
| --- | --- | --- |
| age group at baseline | UK Biobank Touchscreen questionnaire at baseline:  Age when attended assessment centre was divided into 6 groups: <45, 45 to <50, 50 to <55, 55 to <60, 60 to <65 and ≥65 years. | 21003 |
| gender | UK Biobank Touchscreen questionnaire at baseline: Male, female. | 31 |
| qualification | UK Biobank Touchscreen questionnaire at baseline:  University graduate: College or University, Other professional qualifications eg nursing, teaching degree  No university degree: A levels/AS levels or equivalent, O levels/GCSEs or equivalent, CSEs or equivalent, NVQ or HND or HNC or equivalent, None of the above | 6138 |
| Townsend Deprivation Index | A score derived from national census data about car ownership, household overcrowding, owner occupation, and unemployment aggregated for postcodes of residence | 189 |
| family history of cancer | UK Biobank Touchscreen questionnaire at baseline: No: None,  Yes: at least one family member had ever been diagnosed with a cancer | 20107; 20110; 20111 |
| physical activity | UK Biobank IPAQ (International Physical Activity Questionnaire) at baseline:  Low: lowest quartile, ≤918 MET.min/week)  Moderate (918-3706 MET.min/week)  High (highest quartile: >3706 MET.min/week) | 22032 |
| smoking status | UK Biobank Touchscreen questionnaire at baseline:  Current, previous, never smoker. | 20116 |
| alcohol consumption | UK Biobank Touchscreen questionnaire at baseline:  Current, previous, never smoker. | 20117 |
| fruit intake | UK Biobank Touchscreen questionnaire at baseline:  <4 servings/day, ≥4 servings/day  (Amount per serving: fresh fruit- 1 piece; dried fruit- 2 pieces) | 1309, 1319 |
| vegetable consumption | UK Biobank Touchscreen questionnaire at baseline:  <4 servings/day, ≥4 servings/day  (Amount per serving: cooked/raw vegetables- 2 heaped tablespoons) | 1289, 1299 |
| red and processed meat consumption | UK Biobank Touchscreen questionnaire at baseline:  < 2.0, 2.0-2.9, 3.0-3.9, ≥4.0 times per week  (Frequency: for beef, pork, and lamb/mutton, using the following coding: “Never” = 0, "Less than once a week" = 0.5, "Once a week" = 1, "2-4 times a week" = 3, "5-6 times a week" = 5.5, "Once or more daily" = 7) | 1349, 1369, 1379, 1389 |
| regular aspirin or ibuprofen use | UK Biobank Touchscreen questionnaire at baseline:  Yes, No. | 6154 |

**Table S4.** Baseline characteristics among 430,036 participants from UK Biobank

| **Characteristics** | **All**  **(N=430,036)** | **Cases**  **(N=5494)** | **Noncases**  **(N=424,542)** |
| --- | --- | --- | --- |
| Age at baseline, mean (SD), years | 56.56 (8.04) | 60.76 (6.55) | 56.50 (8.04) |
| Gender (%) |  |  |  |
| Male | 199520 (46.40) | 3323 (60.48) | 196197 (46.21) |
| Female | 230516 (53.60) | 2171 (39.52) | 228345 (53.79) |
| Townsend Deprivation Index, mean (SD) | -1.46 (2.99) | -1.39 (3.06) | -1.46 (2.99) |
| Qualification (%) |  |  |  |
| University graduate | 160149 (37.24) | 1815 (33.04) | 158334 (37.30) |
| No university degree | 265719 (61.79) | 3600 (65.53) | 262119 (61.74) |
| Family history of cancer (%) |  |  |  |
| Yes | 152731 (35.52) | 2190 (39.86) | 150541 (35.46) |
| No | 269519 (62.67) | 3200 (58.25) | 266319 (62.73) |
| Smoking status (%) |  |  |  |
| Never | 232372 (54.04) | 2377 (43.27) | 229995 (54.17) |
| Previous | 151175 (35.15) | 2440 (44.41) | 148735 (35.03) |
| Current | 45001 (10.46) | 656 (11.94) | 44345 (10.45) |
| Alcohol consumption (%) |  |  |  |
| Never | 13673 (3.18) | 183 (3.33) | 13490 (3.18) |
| Previous | 14683 (3.41) | 236 (4.30) | 14447 (3.40) |
| Current | 401302 (93.32) | 5067 (92.23) | 396235 (93.33) |
| Physical activity (%) |  |  |  |
| Low | 64861 (15.08) | 896 (16.31) | 63965 (15.07) |
| Moderate | 142302 (33.09) | 1788 (32.54) | 140514 (33.10) |
| High | 142143 (33.05) | 1775 (32.31) | 140368 (33.06) |
| Vegetable consumption (servings/day, %) |  |  |  |
| <4 | 374174 (87.01) | 4806 (87.48) | 369368 (87.00) |
| ≥4 | 55862 (12.99) | 688 (12.52) | 55174 (13.00) |
| Fruit intake (servings/day, %) |  |  |  |
| <4 | 336967 (78.36) | 4314 (78.52) | 332653 (78.36) |
| ≥4 | 93069 (21.64) | 1180 (21.48) | 91889 (21.64) |
| Red and processed meat (times per week, %) |  |  |  |
| < 2.0 | 58056 (13.50) | 551 (10.03) | 57505 (13.55) |
| 2.0-2.9 | 124985 (29.06) | 1487 (27.07) | 123498 (29.09) |
| 3.0-3.9 | 65736 (15.29) | 828 (15.07) | 64908 (15.29) |
| ≥4.0 | 176808 (41.11) | 2564 (46.67) | 174244 (41.04) |
| Regular aspirin/ibuprofen use (%) |  |  |  |
| No | 309010 (71.86) | 3832 (69.75) | 305178 (71.88) |
| Yes | 116298 (27.04) | 1579 (28.74) | 114719 (27.02) |

SD, standard deviation

**Table S5.** Baseline characteristics among site-specific gastrointestinal cancer

| **Characteristics** | **Esophageal cancer (N=782)** |  | **Gastric cancer**  **(N=516)** |  | **Colorectal cancer (N=4205)** |
| --- | --- | --- | --- | --- | --- |
| Age at baseline, mean (SD), years | 61.39 (5.87) |  | 61.37 (6.62) |  | 60.57 (6.65) |
| Gender (%) |  |  |  |  |  |
| Male | 575 (73.53) |  | 353 (68.41) |  | 2402 (57.12) |
| Female | 207 (26.47) |  | 163 (31.59) |  | 1803 (42.88) |
| Townsend Deprivation Index, mean (SD) | -1.14 (3.19) |  | -0.90 (3.26) |  | -1.50 (3.01) |
| Qualification |  |  |  |  |  |
| University graduate | 240 (30.69) |  | 139 (26.94) |  | 1439 (34.22) |
| No university degree | 536 (68.54) |  | 366 (70.93) |  | 2704 (64.30) |
| Family history of cancer |  |  |  |  |  |
| Yes | 296 (37.85) |  | 188 (36.43) |  | 1709 (40.64) |
| No | 465 (59.46) |  | 316 (61.24) |  | 2424 (57.65) |
| Smoking status |  |  |  |  |  |
| Never | 250 (31.97) |  | 198 (38.37) |  | 1933 (45.97) |
| Previous | 376 (48.47) |  | 228 (44.19) |  | 1834 (43.61) |
| Current | 149 (19.05) |  | 85 (16.47) |  | 426 (10.13) |
| Alcohol consumption |  |  |  |  |  |
| Never | 30 (3.84) |  | 18 (3.49) |  | 135 (3.21) |
| Previous | 47 (6.01) |  | 31 (6.01) |  | 158 (3.76) |
| Current | 703 (89.90) |  | 466 (90.31) |  | 3907 (92.91) |
| Physical activity (%) |  |  |  |  |  |
| Low | 142 (18.16) |  | 97 (18.80) |  | 660 (15.70) |
| Moderate | 238 (30.43) |  | 160 (31.01) |  | 1393 (33.13) |
| High | 246 (31.46) |  | 171 (33.14) |  | 1361 (32.37) |
| Vegetable consumption (servings/day) |  |  |  |  |  |
| <4 | 687 (87.85) |  | 453 (87.79) |  | 3675 (87.40) |
| ≥4 | 95 (12.15) |  | 63 (12.21) |  | 530 (12.60) |
| Fruit intake (servings/day) |  |  |  |  |  |
| <4 | 633 (80.95) |  | 396 (76.74) |  | 3290 (78.24) |
| ≥4 | 149 (19.05) |  | 120 (23.26) |  | 915 (21.76) |
| Red and processed meat(times per week) |  |  |  |  |  |
| < 2.0 | 80 (10.23) |  | 47 (9.11) |  | 424 (10.08) |
| 2.0-2.9 | 200 (25.58) |  | 134 (25.97) |  | 1155 (27.47) |
| 3.0-3.9 | 104 (13.302) |  | 76 (14.73) |  | 649 (15.43) |
| ≥4.0 | 387 (49.49) |  | 251 (48.64) |  | 1932 (45.95) |
| Regular aspirin/ibuprofen use |  |  |  |  |  |
| No | 535 (68.41) |  | 339 (65.70) |  | 2962 (70.44) |
| Yes | 232 (29.67) |  | 169 (32.75) |  | 1183 (28.13) |
| MetS |  |  |  |  |  |
| No | 474 (60.61) |  | 303 (58.72) |  | 2799 (66.56) |
| Yes | 308 (39.39) |  | 213 (41.28) |  | 1406 (33.44) |
| Waist circumference, Mean (SD), cm | 97.64 (14.78) |  | 96.66 (14.27) |  | 93.74 (13.64) |
| SBP, Mean (SD), mm Hg | 144 (18.58) |  | 142 (18.84) |  | 142 (18.71) |
| DBP, Mean (SD), mm Hg | 84 (10.29) |  | 83 (10.09) |  | 83 (9.93) |
| Triglycerides, Mean (SD), mmol/L | 1.97 (1.05) |  | 1.95 (1.11) |  | 1.87 (1.05) |
| HDL, Mean (SD), mmol/L | 1.33 (0.35) |  | 1.33 (0.33) |  | 1.41(0.36) |
| HbA1c, Mean (SD), mmol/L | 38.19(8.15) |  | 37.93 (7.43) |  | 36.86 (6.89) |
| Polygenic Risk Score (%) |  |  |  |  |  |
| Low | 123 (15.87) |  | 95 (18.70) |  | 468 (11.14) |
| Intermediate | 467 (60.26) |  | 306 (60.24) |  | 2381 (56.66) |
| High | 185 (23.87) |  | 107 (21.06) |  | 1353 (32.20) |

SD, standard deviation; MetS, metabolic syndrome; SBP, systolic blood pressure; DBP, diastolic blood pressure; HDL, high density lipoproteins; HbA1c, glycated haemoglobin.

The GI-PRS was categorized into low (bottom quintile), intermediate (quintiles 2-4) and high (top quintile) genetic risk.

**Table S6.** Association between metabolic syndrome components and risk of gastrointestinal cancer

| **MetS component** | **N Cases/Person-years** | | **Model 1**^a^ | | **Model 2**^b^ | | **Model 3**^c^ | |
| --- | --- | --- | --- | --- | --- | --- | --- | --- |
|  | **No (ref.)** | **Yes** | **HR (95% CI)** | ***P* value** | **HR (95% CI)** | ***P* value** | **HR (95% CI)** | ***P* value** |
| Central obesity | 3430/3152682 | 2064/1370512 | **1.30(1.23, 1.37)** | **2.95×10^-20^** | **1.27(1.20, 1.34)** | **1.14×10^-16^** | **1.21(1.14, 1.28)** | **1.84×10^-10^** |
| Hypertension | 1010/1285274 | 4484/3237920 | **1.16(1.08, 1.24)** | **5.32×10^-05^** | **1.16(1.08, 1.24)** | **4.49×10^-05^** | **1.11(1.03, 1.19)** | **0.0062** |
| Hypertriglyceridemia | 2323/2397051 | 3171/2126143 | **1.14(1.08, 1.21)** | **2.93×10^-06^** | **1.17(1.06, 1.18)** | **0.0001** | 1.04(0.98, 1.10) | 0.2469 |
| Dyslipidemia | 4464/3751205 | 1030/771988 | **1.19(1.11, 1.27)** | **7.66×10^-07^** | **1.16(1.08, 1.24)** | **1.89×10^-05^** | **1.08(1.01, 1.16)** | **0.0323** |
| Hyperglycemia | 4864/4206635 | 630/316559 | **1.28(1.18, 1.39)** | **7.41×10^-09^** | **1.24(1.14, 1.35)** | **6.81×10^-07^** | **1.14(1.05, 1.25)** | **0.0028** |

aModel 1: adjusted for age group, gender, qualification, Townsend Deprivation Index, family history of cancer

bModel 2: additionally adjusted for smoking status, alcohol consumption, physical activity, fruit intake, vegetable consumption, red and processed meat consumption and regular aspirin or ibuprofen use

cModel 3: additionally adjusted for MetS components

MetS, metabolic syndrome; HR, hazard ratio; CI, confidence interval

**Table S7.** Association between the level of each metabolic syndrome component and gastrointestinal cancer risk

| **MetS component** | **Model 1**^a^ | | **Model 2**^b^ | | **Model 3**^c^ | |
| --- | --- | --- | --- | --- | --- | --- |
|  | **HR (95%CI)** | ***P* value** | **HR (95%CI)** | ***P* value** | **HR (95%CI)** | ***P* value** |
| Waist circumference (cm) | **1.19(1.16, 1.23)** | **2.71×10^-32^** | **1.18(1.14, 1.22)** | **5.17×10^-27^** | **1.16(1.12, 1.20)** | **2.00×10^-18^** |
| SBP (mm Hg) | **1.07(1.04, 1.10)** | **2.11×10^-06^** | **1.07(1.04, 1.10)** | **9.48×10^-07^** | **1.07(1.03, 1.11)** | **0.0002** |
| DBP (mm Hg) | **1.05(1.02, 1.07)** | **0.0009** | **1.05(1.02, 1.08)** | **0.0006** | 0.97(0.94, 1.01) | 0.1368 |
| Triglycerides (mmol/L) | **1.07(1.05, 1.10)** | **1.44×10^-08^** | **1.06(1.03, 1.09)** | **3.34×10^-06^** | 1.02(0.99, 1.05) | 0.1138 |
| HDL (mmol/L) | **0.94(0.91, 0.97)** | **5.95×10^-05^** | **0.95(0.92, 0.98)** | **0.0008** | 1.01(0.97, 1.04) | 0.6680 |
| HbA1c (mmol/mol) | **1.06(1.04, 1.08)** | **1.51×10^-14^** | **1.05(1.04, 1.07)** | **1.64×10^-10^** | **1.03(1.01, 1.06)** | **0.0015** |

Hazard ratio corresponds to one standard deviation increase in MetS components level

aModel 1: adjusted for age group, gender, qualification, Townsend Deprivation Index, family history of cancer

bModel 2: additionally adjusted for smoking status, alcohol consumption, physical activity, fruit intake, vegetable consumption, red and processed meat consumption and regular aspirin or ibuprofen use

cModel 3: additionally adjusted for MetS components level

MetS, metabolic syndrome; SBP, systolic blood pressure; DBP, diastolic blood pressure; HDL, high density lipoproteins; HR, hazard ratio; CI, confidence interval.

**Table S8.** Association between metabolic syndrome and risk of site-specific cancers of gastrointestinal cancer

| **Outcome**^a^ | **N Cases/Person-years** | **Model 1**^b^ | | **Model 2**^c^ | |
| --- | --- | --- | --- | --- | --- |
|  |  | **HR (95%CI)** | ***P* value** | **HR (95%CI)** | ***P* value** |
| Esophageal cancer |  |  |  |  |  |
| No MetS | 468/3389590 | Ref. | Ref. | Ref. | Ref. |
| MetS | 307/1151211 | **1.48(1.28, 1.72)** | **1.09×10^-07^** | **1.41(1.22, 1.63)** | **4.22×10^-06^** |
| Gastric cancer |  |  |  |  |  |
| No MetS | 298/3388952 | Ref. | Ref. | Ref. | Ref. |
| MetS | 210/1150828 | **1.60(1.34, 1.91)** | **3.04×10^-07^** | **1.53(1.28, 1.83)** | **3.41×10^-06^** |
| Colorectal cancer |  |  |  |  |  |
| No MetS | 2798/3378293 | Ref. | Ref. | Ref. | Ref. |
| MetS | 1404/1145800 | **1.24 (1.16, 1.32)** | **8.57×10^-11^** | **1.22(1.15, 1.31)** | **2.23×10^-09^** |

^a^Excluded cases with multi-site tumors

^b^Model 1: adjusted for age group, gender, qualification, Townsend Deprivation Index, family history of cancer

^c^Model 2: additionally adjusted for smoking status, alcohol consumption, physical activity, fruit intake, vegetable consumption. Models for colorectal cancer additionally adjusted for red and processed meat consumption and regular aspirin or ibuprofen use.

MetS, metabolic syndrome; HR, hazard ratio; CI, confidence interval

**Table S9.** Sensitive analysis for gastrointestinal cancer across metabolic syndrome and its components with the definition of obesity in metabolic status according to BMI

| **MetS** | **N Cases/Person-years** | | **Model 1**a | | **Model 2**b | | **Model 3**c | |
| --- | --- | --- | --- | --- | --- | --- | --- | --- |
|  | **No (ref.)** | **Yes** | **HR (95% CI)** | ***P* value** | **HR (95% CI)** | ***P* value** | **HR (95% CI)** | ***P* value** |
| MetS | 3776/3483979 | 1718/1039216 | **1.26(1.19, 1.34)** | **2.19×10^-15^** | **1.24(1.17, 1.31)** | **1.04×10^-12^** |  |  |
| MetS component |  |  |  |  |  |  |  |  |
| Obesity^d^ | 3897/3428507 | 1575/1081149 | **1.22(1.15, 1.29)** | **5.04×10^-11^** | **1.20(1.13, 1.27)** | **2.90×10^-09^** | **1.13(1.06, 1.21)** | **9.51×10^-05^** |
| Hypertension | 1010/1285274 | 4484/3237920 | **1.16(1.08, 1.24)** | **5.32×10^-05^** | **1.16(1.08, 1.24)** | **4.49×10^-05^** | **1.12(1.04, 1.20)** | **0.0023** |
| Hypertriglyceridemia | 2323/2397051 | 3171/2126143 | **1.14(1.08, 1.21)** | **2.93×10^-06^** | **1.12(1.06, 1.18)** | **0.0001** | 1.05(0.99, 1.11) | 0.1185 |
| Dyslipidemia | 4464/3751205 | 1030/771988 | **1.19(1.11, 1.27)** | **7.66×10^-07^** | **1.16(1.08, 1.24)** | **1.89×10^-05^** | **1.10(1.02, 1.18)** | **0.0130** |
| Hyperglycemia | 4864/4206635 | 630/316559 | **1.28(1.18, 1.39)** | **7.41×10^-09^** | **1.24(1.14, 1.35)** | **6.81×10^-07^** | **1.16(1.07, 1.27)** | **0.0006** |
| MetS, metabolic syndrome; HR, hazard ratio; CI, confidence interval | | | | | | | | |

^a^Model 1: adjusted for age group, gender, qualification, Townsend Deprivation Index, family history of cancer

^b^Model 2: additionally adjusted for smoking status, alcohol consumption, physical activity, fruit intake, vegetable consumption, red and processed meat consumption and regular aspirin or ibuprofen use

^c^Model 3: additionally adjusted for MetS components

^d^Obesity is defined as BMI >30 kg/m^2^

**Table S10.** Sensitive analysis for gastrointestinal cancer across metabolic syndrome and components after censoring cases within first two years of follow up

| **MetS** | **N Cases/Person-years** | | **Model 1**^a^ | | **Model 2**^b^ | | **Model 3**^c^ | |
| --- | --- | --- | --- | --- | --- | --- | --- | --- |
|  | **No (ref.)** | **Yes** | **HR (95% CI)** | ***P* value** | **HR (95% CI)** | ***P* value** | **HR (95% CI)** | ***P* value** |
| MetS | 3234/3377245 | 1744/1145178 | **1.31(1.24, 1.39)** | **2.37×10^-19^** | **1.29(1.21, 1.36)** | **2.80×10^-16^** |  |  |
| MetS component |  |  |  |  |  |  |  |  |
| Central obesity | 3098/3152185 | 1880/1370239 | **1.31(1.24, 1.39)** | **5.14×10^-20^** | **1.28(1.21, 1.36)** | **9.83×10^-17^** | **1.23(1.15, 1.31)** | **6.39×10^-11^** |
| Hypertension | 923/1285146 | 4055/3237277 | **1.15(1.07, 1.24)** | **0.0002** | **1.16(1.07, 1.24)** | **0.0001** | **1.10(1.02, 1.19)** | **0.0121** |
| Hypertriglyceridemia | 2118/2396745 | 2860/2125679 | **1.13(1.07, 1.20)** | **2.95×10^-05^** | **1.11(1.05, 1.18)** | **0.0006** | 1.03(0.96, 1.09) | 0.4317 |
| Dyslipidemia | 4047/3750584 | 931/771839 | **1.19(1.10, 1.27)** | **3.18×10^-06^** | **1.16(1.08, 1.25)** | **5.06×10^-05^** | **1.08(1.00, 1.16)** | **0.0452** |
| Hyperglycemia | 4409/4205954 | 569/316469 | **1.28(1.18, 1.40)** | **2.67×10^-08^** | **1.25(1.14, 1.36)** | **1.37×10^-06^** | **1.15(1.05, 1.26)** | **0.0034** |

^a^Model 1: adjusted for age group, gender, qualification, Townsend Deprivation Index, family history of cancer

^b^Model 2: additionally adjusted for smoking status, alcohol consumption, physical activity, fruit intake, vegetable consumption, red and processed meat consumption and regular aspirin or ibuprofen use

^c^Model 3: additionally adjusted for MetS components

MetS, metabolic syndrome; HR, hazard ratio; CI, confidence interval

**Table S11.** Sensitive analysis for gastrointestinal cancer across metabolic syndrome and components with unimputed data

| **MetS** | **N Cases/Person-years** | | **Model 1**^a^ | | **Model 2**^b^ | | **Model 3**^c^ | |
| --- | --- | --- | --- | --- | --- | --- | --- | --- |
|  | **No (ref.)** | **Yes** | **HR (95% CI)** | ***P* value** | **HR (95% CI)** | ***P* value** | **HR (95% CI)** | ***P* value** |
| MetS | 3606/3405621 | 1888/1117573 | **1.30(1.23, 1.38)** | **4.01×10^-20^** | **1.27(1.20, 1.35)** | **1.39×10^-15^** |  |  |
| MetS component |  |  |  |  |  |  |  |  |
| Central obesity | 3418/3145584 | 2064/1370512 | **1.27(1.23, 1.37)** | **2.89×10^-20^** | **1.26(1.19, 1.34)** | **5.57×10^-16^** | **1.22(1.15, 1.31)** | **2.78×10^-09^** |
| Hypertension | 1001/1275395 | 4314/3063195 | **1.17(1.09, 1.25)** | **2.68×10^-05^** | **1.16(1.08, 1.24)** | **7.22×10^-05^** | **1.09(1.00, 1.18)** | **0.0426** |
| Hypertriglyceridemia | 2107/2206539 | 3171/2126143 | **1.15(1.09, 1.22)** | **1.33×10^-06^** | **1.12(1.06, 1.19)** | **0.0002** | 1.03(0.96, 1.10) | 0.4172 |
| Dyslipidemia | 3790/3175647 | 1030/771988 | **1.19(1.11, 1.27)** | **1.30×10^-06^** | **1.16(1.08, 1.25)** | **3.87×10^-05^** | 1.07(0.99, 1.16) | 0.0873 |
| Hyperglycemia | 4580/3972472 | 630/316559 | **1.28(1.18, 1.40)** | **6.21×10^-09^** | **1.26(1.16, 1.37)** | **1.88×10^-07^** | **1.15(1.04, 1.26)** | **0.0064** |

^a^Model 1: adjusted for age group, gender, qualification, Townsend Deprivation Index, family history of cancer

^b^Model 2: additionally adjusted for smoking status, alcohol consumption, physical activity, fruit intake, vegetable consumption, red and processed meat consumption and regular aspirin or ibuprofen use

^c^Model 3: additionally adjusted for MetS components

MetS, metabolic syndrome; HR, hazard ratio; CI, confidence interval

**Table S12.** Sensitive analysis for GI cancer risk across different risk levels of reconstructed GI-PRS after standardizing the mean of each site-specific cancer PRS to 1.

| **Genetic risk group** | **N cases / Person-years** | **HR (95% CI) ^a^** | ***P* value ^a^** |
| --- | --- | --- | --- |
| Per SD |  | 1.54(1.48, 1.60) | 4.42×10^-109^ |
| Low (Q1) | 717/907109 | Ref. | Ref. |
| Intermediate (Q2-Q4) | 3160/2714898 | 1.47(1.36, 1.60) | 1.08×10^-20^ |
| High (Q5) | 1617/901187 | 2.27(2.08, 2.48) | 1.79×10^-74^ |
| *P* value for trend |  |  | 6.58×10^-83^ |

^a^Adjusted for age group, gender, qualification, Townsend Deprivation Index, family history of cancer, smoking status, alcohol consumption and the top 10 genetic principal components.

**Table S13.** Association between the number of high genetic risk for site-specific gastrointestinal cancers and overall gastrointestinal cancer

| **No. of high genetic risk for site-specific GI cancers** | **Genetic risk group of GI cancer** | | | | |
| --- | --- | --- | --- | --- | --- |
|  | **Low**  **(N cases, %)** | **Intermediate**  **(N cases, %)** | **High**  **(N cases, %)** | **HR (95% CI)** | ***P* value** |
| 0 | 531 (74.16) | 1941 (61.68) | 36 (2.22) | Ref. | Ref. |
| 1 | 176 (24.58) | 1074 (34.13) | 981 (60.48) | **1.25 (1.18, 1.32)** | **1.79×10^-14^** |
| 2 | 9 (1.26) | 132 (4.19) | 536 (33.05) | **1.59 (1.46, 1.73)** | **1.02×10^-26^** |
| 3 | 0 (0.00) | 0 (0.00) | 69 (4.25) | **1.98 (1.56, 2.52)** | **2.18×10^-08^** |
| *P* trend |  |  |  |  | **2.82×10^-35^** |

Models adjusted for age group, gender, qualification, Townsend Deprivation Index, family history of cancer, smoking status, alcohol consumption and the top 10 genetic principal components.

**Table S14.** Association between metabolic syndrome within each genetic risk category and risk of site-specific gastrointestinal cancers

| **Outcome**^a^ | **Low genetic risk** | |  | **Intermediate genetic risk** | |  | **High genetic risk** | |
| --- | --- | --- | --- | --- | --- | --- | --- | --- |
|  | **No MetS** | **MetS** |  | **No MetS** | **MetS** |  | **No MetS** | **MetS** |
| Esophageal cancer |  |  |  |  |  |  |  |  |
| No. of cases/Person years | 75/679602 | 48/228308 |  | 283/2035203 | 184/689447 |  | 110/674784 | 75/233455 |
| Hazard ratio(95% CI) | Ref. | 1.39(0.96, 2.01) |  | Ref. | **1.41(1.17, 1.70)** |  | Ref. | **1.42(1.05, 1.91)** |
| *P* value |  | 0.0821 |  |  | **0.0004** |  |  | **0.0223** |
| Gastric cancer |  |  |  |  |  |  |  |  |
| No. of cases/Person years | 58/719088 | 37/243093 |  | 178/2065106 | 128/702524 |  | 62/604757 | 45/205210 |
| Hazard ratio(95% CI) | Ref. | 1.41(0.93, 2.15) |  | Ref. | **1.57(1.24, 1.97)** |  | Ref. | **1.56(1.05, 2.31)** |
| *P* value |  | 0.1056 |  |  | **0.0002** |  |  | **0.0262** |
| Colorectal cancer |  |  |  |  |  |  |  |  |
| No. of cases/Person years | 321/676592 | 147/231185 |  | 1578/2027174 | 803/688628 |  | 899/674525 | 454/225986 |
| Hazard ratio(95% CI) | Ref. | 1.08(0.88, 1.32) |  | Ref. | **1.24(1.14, 1.35)** |  | Ref. | **1.25(1.12, 1.41)** |
| *P* value |  | 0.4821 |  |  | **1.31×10^-06^** |  |  | **0.0001** |

^a^Excluded cases with multi-site tumors

Models adjusted for age group, gender, qualification, Townsend Deprivation Index, family history of cancer, smoking status, alcohol consumption, physical activity, fruit intake, vegetable consumption and the top 10 genetic principal components. Models for colorectal cancer additionally adjusted for red and processed meat consumption and regular aspirin or ibuprofen use

MetS, metabolic syndrome; HR, hazard ratio; CI, confidence interval.
